# Jing-Yin-Gu-Biao formula protects mice from postinfluenza *Staphylococcus aureus* infection by ameliorating acute lung injury and improving hypercoagulable state via inhibiting NETosis

**DOI:** 10.3389/fimmu.2025.1567522

**Published:** 2025-03-11

**Authors:** Biao Lei, Jingwen Mu, Guihua Xu, Xiaodong Yang, Wenbo Huang, Liang Hu, Dan Liu, Ting Cheng, Yuhe Ma, Lirong Xu, Qiankun Liang, Yuan Lin, Linqiong Zhou, Chunxian Zhou, Wei Zhang, Yuejuan Zheng

**Affiliations:** ^1^ The Research Center for Traditional Chinese Medicine, Shanghai Institute of Infectious Diseases and Biosecurity, School of Integrative Medicine, Shanghai University of Traditional Chinese Medicine, Shanghai, China; ^2^ Center for Traditional Chinese Medicine and Immunology Research, School of Integrative Medicine, Shanghai University of Traditional Chinese Medicine, Shanghai, China; ^3^ Department of Pulmonary Diseases, ShuGuang Hospital Affiliated to Shanghai University of Traditional Chinese Medicine, Shanghai, China; ^4^ State Key Laboratory of Respiratory Disease, National Clinical Research Center for Respiratory Disease, Guangzhou Institute of Respiratory Health, the First Affiliated Hospital of Guangzhou Medical University, Guangzhou, Guangdong, China; ^5^ Shuguang Hospital, Shanghai Institute of Infectious Diseases and Biosecurity, Shanghai University of Traditional Chinese Medicine, Shanghai, China; ^6^ Shanghai University of Traditional Chinese Medicine Epidemic Research Center, Shanghai, China; ^7^ Shanghai Baoshan Hospital of Integrated Traditional Chinese and Western Medicine, Respiratory and Critical Care Medicine, Shanghai, China

**Keywords:** Jing-Yin-Gu-Biao formula, postinfluenza *Staphylococcus aureus* infection, neutrophil extracellular trap, platelet activation, ERK/ROS axis

## Abstract

**Background:**

Jing-Yin-Gu-Biao formula (JYGBF) is a Chinese medicine derived from Yupingfeng power, Huoxiangzhengqi powder and Yinqiao powder, and has been widely used to treat acute respiratory infections. This study aims to observe the effects of JYGBF against postinfluenza *Staphylococcus aureus* (*S. aureus*) infection.

**Purpose and study design:**

A mouse model of secondary *S. aureus* infection following PR8 infection was established to evaluate the protective effects of JYGBF against postinfluenza *Staphylococcus aureus* (*S. aureus*) infection and related mechanisms were validated *in vivo* and *in vitro*.

**Results:**

The administration of JYGBF significantly ameliorated acute lung injury (ALI) and inhibited overactivated inflammatory response (MIP-2, IL-6, etc.) in mice with postinfluenza *S. aureus* infection. Single cell RNA-sequencing (scRNA-seq) data indicated that neutrophils had the highest cytokine score in lungs and JYGBF inhibited neutrophil chemotaxis, reactive oxygen species (ROS) biosynthesis and ERK1/2 cascades in neutrophils. Meanwhile, JYGBF inhibited the formation of neutrophil extracellular traps (NETs) in lungs, which is characterized by the production of ROS, peptidyl arginine deiminase 4 (PAD4), citrullinated histone H3 (CitH3), myeloperoxidase (MPO), neutrophil elastase (NE), S100A8/A9 and MPO-CitH3 colocalization. Moreover, JYGBF decreased platelet counts and the expression of its activated markers (CD62P and αIIbβ3) accompanied by the drop of fibrinogen (FIB) and fibrin degradation product (FDP), accounting for alleviating hypercoagulable state. JYGBF inhibited ERK1/2 phosphorylation in neutrophils and in lungs of infected mice. Acacetin, a critical compound from JYGBF, inhibited NET formation via downregulating ERK/ROS axis.

**Conclusions:**

These results indicated that JYGBF inhibited NET formation and overactivated inflammatory response by suppressing ERK/ROS axis in neutrophils, thereby mitigating ALI and improving the hypercoagulable state during postinfluenza *S. aureus* infection. JYGBF could be considered a potent therapeutic agent for the prevention and treatment of postinfluenza bacterial infection.

## 1 Introduction

Influenza virus infection leads to nearly 3 to 5 million severe cases and 290 to 650 thousand deaths worldwide annually (1). Hospitalized patients of respiratory viral infections complicated with bacterial pathogens are associated with severe disease and higher mortality (2). Up to 95% and 50% of severe or fatal cases were complicated by bacterial infections during the 1918 and 2009 influenza pandemics, respectively (3–6). A meta-analysis containing nearly 50,000 hospitalized flu patients showed that coinfection with bacteria in flu patients is the deadliest risk factor for mortality, which can increase the risk of death by 3.4 times in patients with influenza (7). *Streptococcus pneumonia* and *Staphylococcus aureus* (*S. aureus*) are the most common pathogens, which account for 30.7% and 30.4% of bacterial infections in flu patients (7). Patients coinfected with influenza virus and *S. aureus* progress rapidly to death with up to 41% fatality rate and have become an intractable clinical problem (8). Antiviral drugs, antibiotics and corticosteroids are primary strategies for the treatment of bacterial coinfection in influenza. However, antibiotic treatment could release bacterial components causing inflammatory responses. Corticosteroid treatment could prolong hospitalization days in the ICU or even increase mortality in patients with flu-associated pneumonia (9). The effects of all antiviral drugs on mortality in severe influenza remain uncertain unless they are taken at the early stage (10). It’s crucial to develop more efficient strategies for the treatment of bacterial infections in influenza.

Coinfection of influenza virus and bacteria could contribute to more severe immune responses and immunopathological damage (11, 12). Different microbial components could be sensed by different pattern recognition receptors, which lead to more hyper-activated signaling pathways and excessive immune responses (13). Neutrophils, one of the most abundant circulating leukocytes, play a pivotal role in combating invading pathogens. However, neutrophils are one of the major drivers of overactivated inflammatory response (12). Activated neutrophils secret high levels of chemokines recruiting more inflammatory cells into infected organs (14). Influenza virus or bacterial infection could activate ERK MAPK signaling pathway and lead to the production of reactive oxygen species (ROS). Peptidyl arginine deiminase 4 (PAD4) and myeloperoxidase (MPO) are subsequently stimulated by ROS, and contribute to chromatin decondensation, leading to the formation of neutrophil extracellular traps (NETs). This process refers to NETosis. In summary, pathogenic stimuli could activate ERK/ROS axis and contribute to the formation of NETs (15). Activated neutrophils and the formation of NETs can contribute to lung tissue damage, platelet activation and immunothrombosis by releasing high levels of S100A8/A9, histones and neutrophil elastase (NE) in acute respiratory infection (16, 17). Disseminated intravascular coagulation (DIC) is a severe syndrome characterized by extensive intravascular coagulation and can lead to organ dysfunction. Clinical studies indicated that the formation of NETs is associated with the occurrence of DIC and mortality in septic patients (18). Therefore, effective strategies are urgently needed to inhibit the overactivated neutrophils and the formation of NETs to prevent tissue damage and severe coagulopathy.

Some Chinese medicine exhibits antiviral, antibacterial and anti-inflammatory effects (19–21). They have been extensively recommended for the treatment of acute respiratory infections such as influenza and COVID-19 in China, and they could shorten the period of symptom recovery and improve the prognosis of the disease (22). Recently, some Chinese medicine such as Liu Shen Wan and Lianhuaqingwen capsules have been found to exert protective effects against postinfluenza *S. aureus* infection by preventing *S. aureus* adherence to epithelial cells (21, 23).

JYGBF, a famous Chinese formula, has been approved by the Shanghai Municipal Drug Administration for the treatment of COVID-19 (No. Z20220008000), and has been widely used to treat acute respiratory infection. Studies have found that JYGBF treatment could shorten the negative conversion time and hospital length of stay in patients with COVID-19 (24, 25). JYGBF is composed of 9 herbs including *Lonicera japonica* Thunb. (Jinyinhua), *Nepeta cataria* L. (Jingjie), *Astragalus propinquus Schischkin* (Huangqi), *Saposhnikovia divaricate* (Fangfeng), *Agastache rugosa* (Huoxiang), *Isatis indigotica* (Banlangen), *Platycodon Grandiflorum* (Jiegeng), *Phragmitis rhizoma* (Lugen) and *Glycyrrhiza glabra* L. (Gancao). *Lonicera japonica, Astragalus propinquus Schischkin* and *Isatis indigotica* possess antiviral activity and exert inhibitory effects on virus-induced inflammatory response (24, 26, 27). Multiple components derived from *Agastache rugosa, Isatis indigotica*, and *Platycodon Grandiflorum* could ameliorate influenza virus-induced pneumonia *in vivo* (28–30). According to the fundamentals of traditional Chinese medicine, postinfluenza bacterial infection refers to being invaded by pathogenic factors such as wind, pathogenic heat, toxic pathogens, etc. From the theory of TCM, JYGBF is supposed to not only clear heat and remove toxins, but reinforce healthy qi to eliminate pathogenic factors. JYGBF might be a promising drug candidate for the prevention and treatment of postinfluenza bacterial infection. However, whether JYGBF could play a protective role against postinfluenza *S. aureus* infection remains unknown.

In this study, data showed that JYGBF could protect mice from postinfluenza *S. aureus* infection by alleviating acute lung injury (ALI) and improving the hypercoagulable state. Furthermore, the therapeutic roles of JYGBF were realized through inhibiting neutrophil chemotaxis, neutrophil-mediated immune response, the formation of NETs and neutrophil-platelet interactions via inhibiting ERK/ROS axis. Acacetin, one of the active components of JYGBF, was shown to inhibit NETosis by decreasing the activation of ERK/ROS axis. Our study suggested that JYGBF exerted protective effects on postinfluenza *S. aureus* infection, which was associated with the inhibition of the chemotaxis of neutrophil and NETosis by suppressing ERK/ROS axis.

## 2 Materials and methods

### 2.1 Formula and reagents

The JYGBF formula is composed of 9 herbs: *Lonicera japonica* 9 g, *Nepeta cataria* 9 g, *Astragalus propinquus Schischkin* 12 g, *Saposhnikovia divaricate* 9 g, *Agastache rugosa* 9 g, *Isatis indigotica* 9 g, *Platycodon Grandiflorum* 6 g, *Phragmitis rhizoma* 15 g, *Glycyrrhiza glabra* 9 g. A total of 87 g raw herbs were processed as granules and packaged into 2 sachets (10 g granules per sachet). The recommended oral dose of JYGBF is 20 g granules per day. For animal experiments, the clinical dose is chosen to be the low dose in this study. The low dose of JYGBF = 20 g/day × 12/60 kg = 4 g/kg/day (The clinical dosage of JYGBF for an adult is 20 g/person/day, the equivalent dose ratio of mouse to human is 12, and the average weight of an adult is supposed to 60 kg). The granules were provided by Shanghai Wanshicheng Pharmaceutical Co. The main components of JYGBF were detected by an ultra-high performance liquid chromatography-quadrupole/Orbitrap high-resolution mass spectrometry (UHPLC-Q-Orbitrap HRMS) in the previous study (31).

JYGBF-containing sera and control sera were prepared as follows. SD rats were orally gavaged with JYGBF (8 g/kg) or the same volume of water once a day for 7 consecutive days. One hour after the last intragastric administration, the rats were culled, and the blood samples were collected. The sera were obtained as previously described (32).

### 2.2 Mice

Specific pathogen-free (SPF) C57BL/6J mice (female, 6-8 weeks old) were obtained from Hunan SJA Laboratory Animal Co. Ltd (Hunan, China) and were kept in a pathogen-free environment with free access to standard food and water. All mice received adaptive feeding for a week and were kept in the facility with controlled humidity (60–80%), temperature (22 ± 1°C) and a 12 h light/dark cycle. All *in vivo* experiments were approved by the Ethics Committee of Guangzhou Medical University (No. 20230543B).

### 2.3 Viruses and bacterial strain

The influenza A/Puerto Rico/8/1934 (H1N1) virus strain (PR8) was kindly provided by Robert G. Webster from St. Jude Children’s Research Hospital. The viruses were reproduced and stored as previously described (32).

*Staphylococcus aureus* (*S. aureus*) (ATCC 29213) was purchased from ATCC and employed to establish a secondary infection model in this study. *S. aureus* was recovered and incubated on agar plates at 37°C for 18 h. The bacteria were cultured in Luria-Bertani (1% Tryptone, 0.5% Yeast Extract, 1% NaCl) broth for 12 h with horizontal shaking at 250 rpm and 37°C subsequently. The concentration of bacteria was diluted to 1 × 10^6^ CFU/50 μL with sterile phosphate-buffered saline (PBS) for the infective experiments in mice.

### 2.4 Animal experiments

Mice were randomly divided into 6 groups including PBS group, postinfluenza *S. aureus* infection group (PR8 + *S. aureus*), oseltamivir treatment group (PR8 + *S. aureus* + OSV, 30 mg/kg), low dose of JYGBF treatment group [PR8 + *S. aureus* + JYGBF (L), 4 g/kg], high dose of JYGBF treatment group [PR8 + *S. aureus* + JYGBF (H), 8 g/kg], the combination of oseltamivir and JYGBF treatment group [PR8 + *S. aureus* + OSV (30 mg/kg) + JYGBF (4 g/kg)]. After being anesthetized with isoflurane, mice were challenged with 0.25 LD_50_ PR8 in the infected group or inhaled with 50 μL PBS in control group on day 0. After being infected with 0.25 LD_50_ PR8 for 5 days, infected mice were inhaled with *S. aureus* (1 × 10^6^ CFU/50 μL) sequentially. For survival experiments, all of the infected mice were administered with JYGBF (8 g/kg and 4 g/kg), OSV (30 mg/kg) or OSV (30 mg/kg) + JYGBF (4 g/kg) once daily for 8 consecutive days. The body weight and clinical symptoms (piloerection, hunchbacked posture, dyspnea, reduced activity and half-closed eyes) were monitored daily for 19 consecutive days. The survival rate was calculated. For other experiments, all of the infected mice were administered with JYGBF (8 g/kg and 4 g/kg), OSV (30 mg/kg) or OSV (30 mg/kg) + JYGBF (4 g/kg) once daily for 6 or 7 days. At 6 or 7 days post infection (dpi), lung tissues were collected and weighed for calculating lung indexes [lung index = (lung weight/body weight) × 100%]. Subsequently, lung tissues were used for further analysis including single cell RNA-sequencing (scRNA-seq), Flow cytometric analysis, pathological observation and immunofluorescence, etc. The lung tissues were homogenized and the supernatants of lung homogenates were used to detect the total protein content by BCA protein assay kits (Pierce, Rockford, IL).

### 2.5 Lung collection and dissociation for scRNA-seq

Five lung samples were randomly selected from each group including PBS group, PR8 + *S. aureus* group, PR8 + *S. aureus* + OSV group, PR8 + *S. aureus* + JYGBF (4 g/kg) group, PR8 + *S. aureus* + OSV + JYGBF group. Single-cell suspensions were prepared using enzymatic and mechanical dissociation. The 10× Genomics platform was utilized to capture cells and synthesize cDNA. ScRNA-seq was carried out on a NovaSeq 6000 platform.

### 2.6 ScRNA-seq data processing

ScRNA-seq data were aligned to the reference genome and gene counts were quantified by Cell Ranger software (v7.0.1) to obtain the UMI matrix, which was further loaded into R (v4.2.2) as Seurat objects and processed with the Seurat package (v4.3.2). Cells with less than 200 genes, more than 5000 genes or a high percentage (> 25%) of UMIs mapped to mitochondrial genes were excluded. Seurat’s SCTransform was used to normalize and scale each sample. The batch effect between different samples was removed using harmony, which was used for further analysis and visualization. Principal component analysis (PCA) was then run on the top 2000 highly variable genes and the top 30 principal components (PCs) were used for cluster analysis. Cell types were annotated using the SingleR package (v1.2.4) and then checked manually.

### 2.7 ScRNA-seq analysis

The cell proportions of different groups and cytokine score were analyzed as previously mentioned (19). The cytokine list was shown in **Supplementary Table 1**. The FindAllMarkers function in Seurat was used to identify differentially expressed genes (DEGs) with default parameters. Marker genes were defined as those with adjusted *p* values less than 0.05 and |fold change| larger than 1.2. Kyoto Encyclopedia of Genes and Genomes (KEGG) and Gene Ontology (GO) was performed with clusterProfiler v3.18.0. Terms were enriched with the nominal *p* value < 0.05 and false discovery rate (q value) < 0.05. Gene set variation analysis (GSVA) was performed to identify significantly enriched genes in each transcriptional dataset, using R package GSVA (version 1.46.0) (https://www.gsea-msigdb.org/gsea/index.jsp) on the Reactome gene set with default parameters, respectively.

### 2.8 Histological analysis

The lung tissues were collected and fixed in 4% paraformaldehyde immediately. After being embedded in paraffin, the lung tissues were sliced into 5 μm thickness sections and stained with hematoxylin and eosin (H&E) reagents. The images were captured with an Axio Imager M2 optical microscope. The histological scores were analyzed as previously described (19).

### 2.9 Immunofluorescence assay

Antibodies were purchased from Abcam (anti-MPO, cat# ab208670; anti-CitH3, cat# ab219407; anti-CD41, cat# ab134131; anti-Ly6G, cat# ab238132) and Record Biological Technology Co. Ltd (DAPI, cat# RC05; goat anti-rabbit secondary antibody, cat# RCA054; goat anti-rat secondary antibody, cat# RCE054). The frozen lung sections were blocked in 5% BSA and stained with primary antibodies overnight at 4°C, followed by incubation with secondary antibodies. Finally, the images were obtained with a multi-channel fluorescence scanner (3D Histech, Panoramic Midi).

For *in vitro* experiments, phorbol 12-myristate 13-acetate (PMA)-stimulated neutrophils with or without acacetin were fixed in 4% paraformaldehyde. Subsequently, the neutrophils were permeabilized by immunostaining permeabilization buffer (cat# P0096, Beyotime) for 10 min, which were then blocked with 5% BSA containing with 0.02% Triton X-100 at room temperature for 30 min. After that, neutrophils were stained with anti-CitH3 (cat# EM30605, HUABIO) overnight at 4°C, followed by incubation with secondary antibodies (cat# 8890S, CST, USA). The nuclei were stained with antifade mounting medium containing DAPI (cat# P0131, Beyotime) for another 10 min. Finally, the images were obtained with a confocal laser scanning microscope (LSM 900, ZEISS) and analyzed by ZEN (version 2012).

### 2.10 Reverse transcription and quantitative real-time PCR

The TRIzol reagents were used to extract the total RNA from the lung tissues. The RNA samples were reversely transcribed into cDNA using PrimeScript™ RT Master Mix (Takara, Dalian, China) and cDNA amplification was performed using SYBR Premix Ex Taq kit (Takara, Dalian, China). The 2^-ΔΔCt^ method was used to analyze data. The sequences of primers are shown in **Supplementary Table 2**.

### 2.11 Flow cytometry

Lung tissues were minced in PBS containing collagenase and DNase I, and digested by gentle MACS Octo with Heaters (Miltenyi Biotec, Germany) to obtain lung single-cell suspensions. Lysed by red blood cell lysis buffer (cat# 555899, BD, USA), the single-cell samples were stained with Zombie (cat# 423101, BioLegend, San Diego, CA, USA) and a mixture of anti-mouse CD45 antibody (cat# 103108, BioLegend, San Diego, CA, USA), anti-mouse CD3 antibody (cat# 100216, BioLegend, San Diego, CA, USA), anti-mouse Ly6G antibody (cat# 127641, BioLegend, San Diego, CA, USA), anti-mouse Ly6C antibody (cat# 128032, BioLegend, San Diego, CA, USA) and anti-mouse F4/80 antibody (cat# 123114, BioLegend, San Diego, CA, USA), sequentially.

### 2.12 Detection of coagulation indexes and platelet markers

Blood samples were obtained from the abdominal aorta of mice on the 7^th^ day after infection. The sera were used to detect fibrinogen (FIB, cat# 10501185900, Mindray) and fibrin degradation product (FDP, cat# Y231220, Mindray) by automated coagulation analyzer (C3100, Mindray). Platelet-rich plasma (PRP) was incubated with or without thrombin (0.1 U/mL) for 30 min and then was incubated with P-selectin (CD62P) and αIIbβ3 integrin (cat# D200, Emfret, Germany) at room temperature for 30 min. Finally, the expression of CD62P and αIIbβ3 was measured by Flow cytometry.

### 2.13 Neutrophil isolation

CD11b^+^Ly6G^+^ neutrophils were negatively isolated from mouse bone marrow using a neutrophil separation kit (cat# 70907-100, BEAVER, Suzhou China). The purity of the neutrophils was analyzed by Flow cytometry.

### 2.14 Scanning electron microscopy

Neutrophils were stimulated with PMA (100 ng/mL) or treated with acacetin for 4 h. After that, neutrophils were fixed with 2.5% glutaraldehyde (Merck, Germany) for 4 h and then washed with PBS 3 times. Neutrophils were then post-fixed by 1% osmium tetroxide (Merck, Germany) and washed with PBS again. Followed being dehydrated by a graded series of ethanol, samples were dried, sputtered with gold particles, and analyzed by scanning electron microscopy (FEI ESEM QUANTA 200).

### 2.15 Detection of ROS

Isolated neutrophils (1 × 10^5^) were plated in a 96-well plate and treated with PMA or acacetin. ROS production was detected by ROS detection assay kits (cat# S0033S, Beyotime). After stimulation for 4 h, neutrophils were incubated with fluorescence probe (DCFH-DA) at 37°C in 5% CO_2_ for 30 min. ROS were detected by EnSight^®^ Multimode Plate Reader (EnSight, Revvity, USA) within 30 min.

### 2.16 Enzyme-linked immunosorbent assay

The lung tissues were homogenized in 1 mL PBS by a tissue grinder (SCIENTZ-48, China) at 60 Hz for 90 s. The samples were centrifuged at 10,000 × g at 4°C for 20 min, and the supernatants of lung homogenates was obtained. The concentrations of MIP-2, IL-6, TNF-α, ICAM-1, IL-10, MPO, S100A8/A9 and CXCL4 were measured by mouse ELISA kits (MIP-2, cat# MM200; IL-6, cat# M6000B; TNF-α, cat# MTA00B; IL-10, cat# M1000B. ICAM-1 and MPO, YEPCOME, Shanghai, China; CXCL4, S100A8 and S100A9, BYabscience, Nanjing, China).

### 2.17 Western blot

Lung tissues were lysed on ice for 30 min with RIPA lysis buffer (cat# P0013B, Beyotime, Shanghai, China) supplemented with protease inhibitor cocktail (Roche, Switzerland) and phosphatase inhibitor cocktail (Roche, Switzerland). Protein concentrations were measured by a BCA protein assay kit (Pierce, USA). Western blots were carried out as previously described (33). The primary antibodies including phospho-p44/42 MAPK (p-ERK1/2, cat# 4370, CST, USA), p44/42 МАРK (ERK1/2, cat# 4695, CST, USA), PAD4 (cat# ab214810, Abcam, UK), MPO (cat# ab208670, Abcam, UK) or β-Actin (cat# 3700, CST, USA) were incubated overnight at 4°C, followed by incubation with the secondary antibodies (anti-rabbit IgG, cat# 7074, CST, USA or anti-mouse IgG cat# 7076, CST, USA) for 1 h.

### 2.18 Statistical analysis

All the data were shown as mean ± SD and analyzed by Prism 8.0 (GraphPad, San Diego, CA, United States). Comparisons between multiple groups were conducted using one-way analysis of variance (one-way ANOVA) with Bonferroni or Dunnett test. The student *t*-test was employed for comparison between PR8 + *S. aureus* + OSV group and PR8 + *S. aureus* + OSV + JYGBF group.

## 3 Result

### 3.1 JYGBF exerts protective effects against postinfluenza *S. aureus* infection *in vivo*

**Figure 1:**
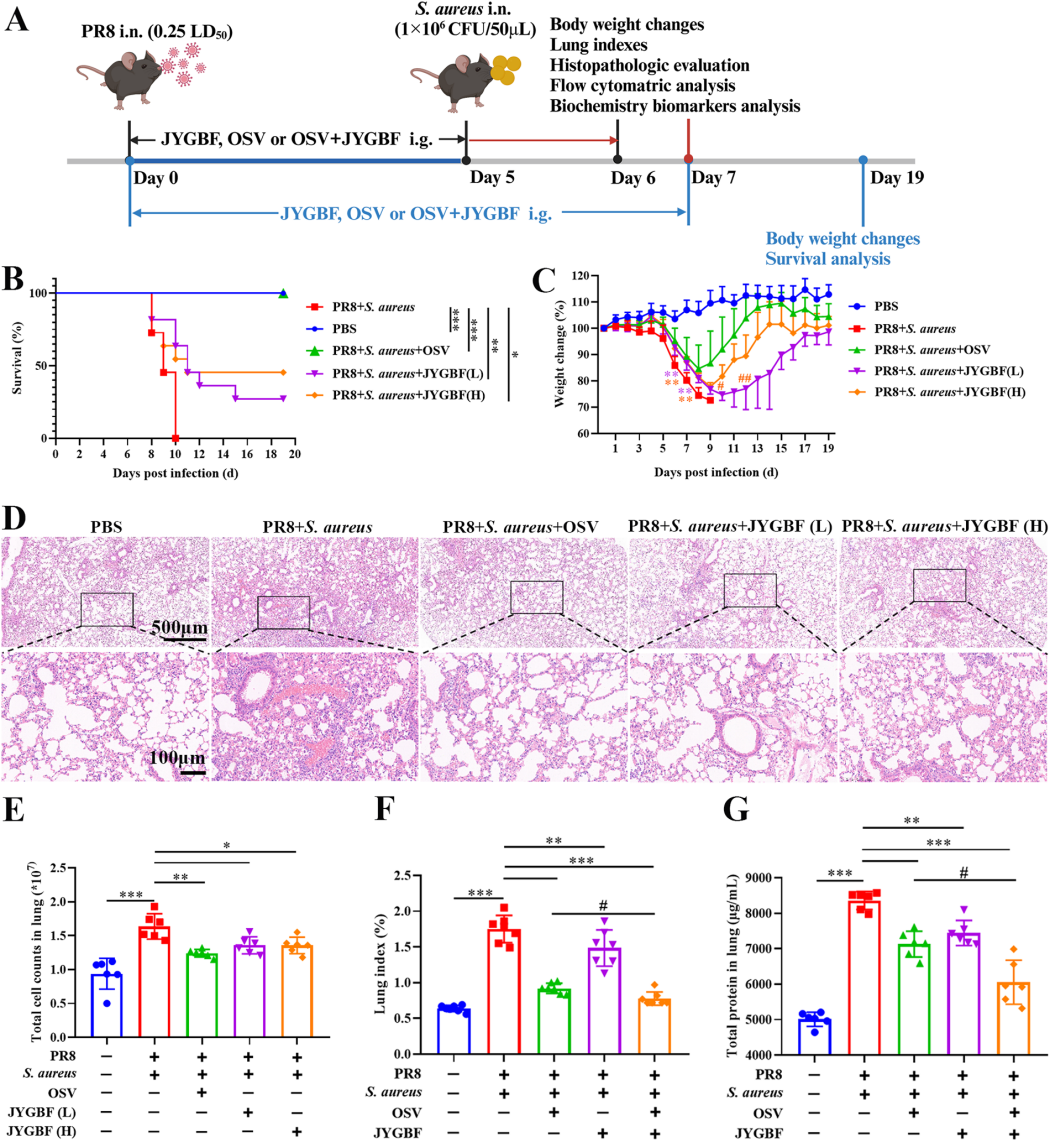
JYGBF exerted protective effects against postinfluenza *S. aureus* infection. **(A)** Study flow chart. **(B)** Survival rate of mice (n=11). **(C)** Body weight changes of mice from 0 to 19 dpi (n=11). **(D)** H&E staining of lungs at 6 dpi. **(E)** Total cell counts in lungs at 6 dpi. **(F)** Lung indexes at 7 dpi (n=7). **(G)** Total protein content of lungs at 7 dpi (n=6). Data are shown as mean ± SD. *, *p* < 0.05; **, *p* < 0.01; ***, *p* < 0.001; *vs*. PR8 + *S. aureus *group. #, *p* < 0.05; ##, *p* < 0.01; *vs*. PR8 + *S. aureus* + OSV group.

To evaluate the effects of JYGBF against postinfluenza *S. aureus* infection, a mouse model of secondary *S. aureus* infection following influenza virus infection was established (**Supplementary Figure S1**). The intranasal infection of *S. aureus* (10^6^ CFU/50 μL, 10^7^ CFU/50 μL and 10^8^ CFU/50 μL) did not cause death in mice (**Supplementary Figures S1A, B**). Though the high dose of *S. aureus* infection led to mild body weight loss at 1 dpi, the lowest dose of *S. aureus* (10^6^ CFU/50 μL) had little impact on the body weight loss at 1 dpi (**Supplementary Figures S1A, B**). Influenza virus infection (0.25 LD_50_) presented a 90.9% survival rate (**Supplementary Figures S1C, D**). The lethal dose of *S. aureus* was optimized to challenge mice at day 5 after PR8 infection (0.25 LD_50_) (**Figure 1A**).

The mice in postinfluenza *S. aureus* infection group all died within 3 days (**Figure 1B**). The clinical equivalent dose of JYGBF [JYGBF (L)], a double dose of clinical dose of JYGBF [JYGBF (H)] and OSV treatment prolonged the survival period of mice and increased the survival rate from 0% to 27.3%, 45.5% and 100%, respectively (**Figure 1B**). Compared with PBS group, the mice in postinfluenza *S. aureus* infection group exhibited a dramatic decrease in body weight loss (**Figure 1C**). JYGBF(L), JYGBF(H) or OSV treatment could significantly attenuate the body weight loss (**Figure 1C**). In JYGBF(H)-treated group, the body weight recovered faster than the JYGBF(L)-treated group at 10 dpi and 12 dpi (**Figure 1C**).

To determine if JYGBF could protect mice against secondary *S. aureus* infection-induced ALI, lung indexes, pathological changes and total cell counts in lungs were analyzed at 6 dpi. Compared with PBS group, secondary *S. aureus* infection led to severe pulmonary lesions. Massive inflammatory cells, congestion and consolidation were observed in the lungs. Different doses of JYGBF and OSV treatment could significantly reduce the infiltration of inflammatory cells and mitigate pathological changes caused by secondary *S. aureus* infection (**Figure 1D**). A significant increase of total cell counts in lungs was caused by secondary infection (**Figure 1E**). Different doses of JYGBF and OSV treatment decreased the total cell numbers in lungs (**Figure 1E**). In recent years, the combined antiviral therapies to treat severe influenza have aroused widespread interest, and a growing number of clinical studies showed that the combined therapy could usually reduce the selection of antiviral resistance mutations, and the frequency of severe cases or complications of infection (34). In our current research, the combination of OSV and JYGBF was set up to determine their synergistic effects against postinfluenza *S. aureus* infection. Postinfluenza *S. aureus* infection resulted in a dramatic increase of lung indexes and total protein content in lungs (**Figures 1F, G**). JYGBF, OSV and the combination of OSV and JYGBF significantly decreased the lung indexes and total protein content (**Figures 1F, G**). Intriguingly, the combination of OSV and JYGBF was superior to OSV treatment in the reduction of inflammatory exudates and protein secretion into lungs (**Figures 1F, G**).

### 3.2 JYGBF relieves clinical symptoms of infected mice

**Figure 2:**
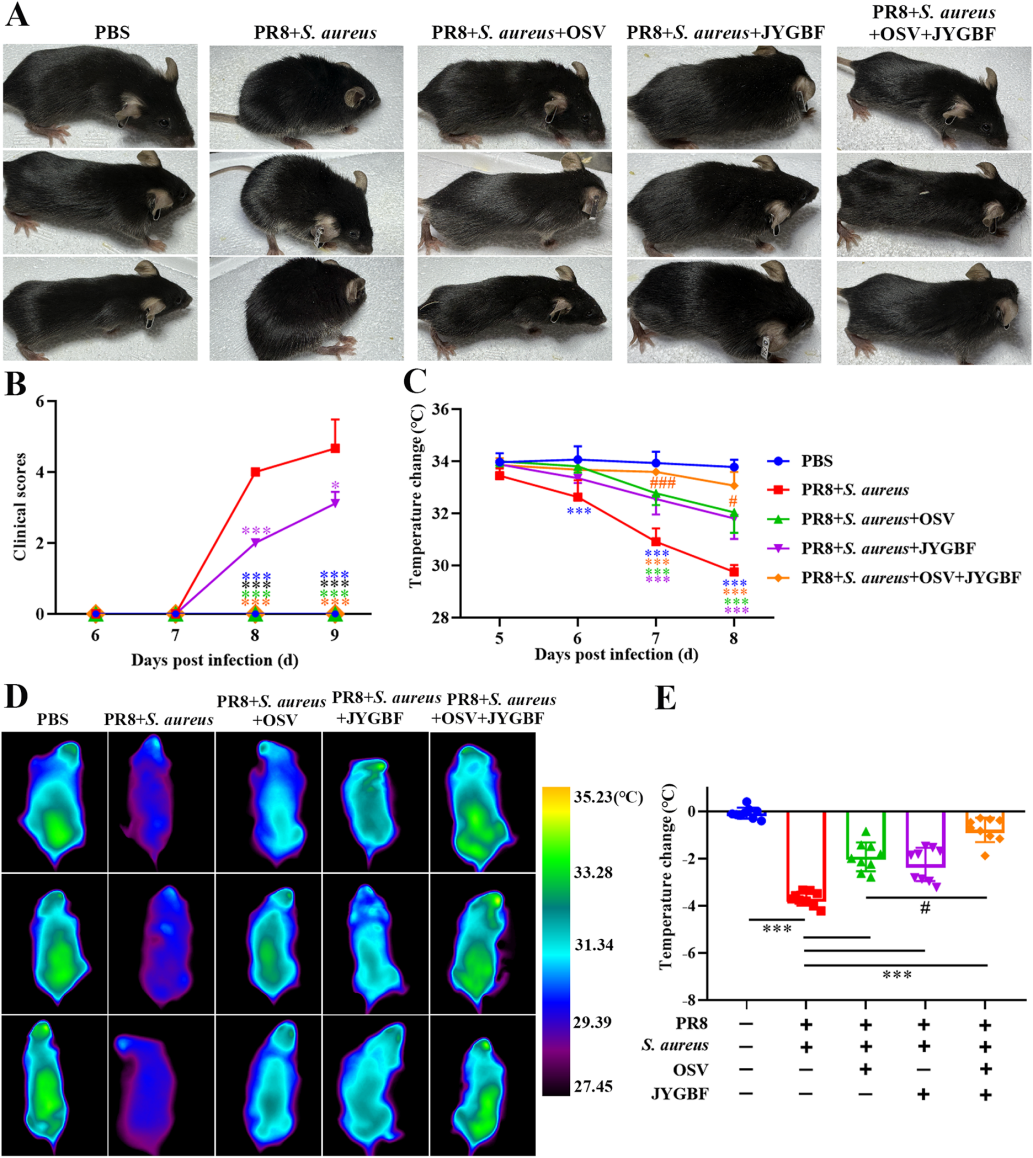
JYGBF relieved symptoms of mice during secondary *S. aureus* infection. **(A)** The appearance of mice at 8 dpi. **(B)** Clinical symptom scores from 6 to 9 dpi (n=9). **(C)** Body temperature changes from 5 to 8 dpi (n=9). **(D)** The body temperature of mice measured by infrared thermometer at 8 dpi. **(E)** Body temperature changes of mice at 8 dpi (n=9). Data are shown as mean ± SD. *, *p* < 0.05; ***, *p* < 0.001; *vs*. PR8 + *S. aureus* group. #, *p* < 0.05; ###, *p* < 0.001; *vs*. PR8 + *S. aureus* + OSV group.

Mild influenza virus (0.25 LD_50_) or bacterial infection (10^6^ CFU/50μL) did not cause any clinical obvious signs (data not shown). However, postinfluenza *S. aureus* infection caused death rapidly with obvious clinical signs including piloerection, hunchbacked posture, breathlessness, reduced movement and half-closed eyes at 8 and 9 dpi (**Figures 2A, B**). Consistent with the protective effects described above, JYGBF, OSV or the combination of OSV and JYGBF could relieve symptoms caused by serious infection (**Figures 2A, B**). In addition, postinfluenza *S. aureus* infection led to a drop in body temperature in mice from 6 to 8 dpi (**Figures 2C–E**). The treatment of JYGBF, OSV and the combined treatment could reverse the reduction of body temperature at 7 and 8 dpi (**Figures 2C–E**). The combined treatment was significantly better than the OSV treatment alone in temperature recovery at 7 and 8 dpi (**Figures 2C–E**).

### 3.3 JYGBF attenuates the excessive systemic inflammation, especially in lungs

**Figure 3:**
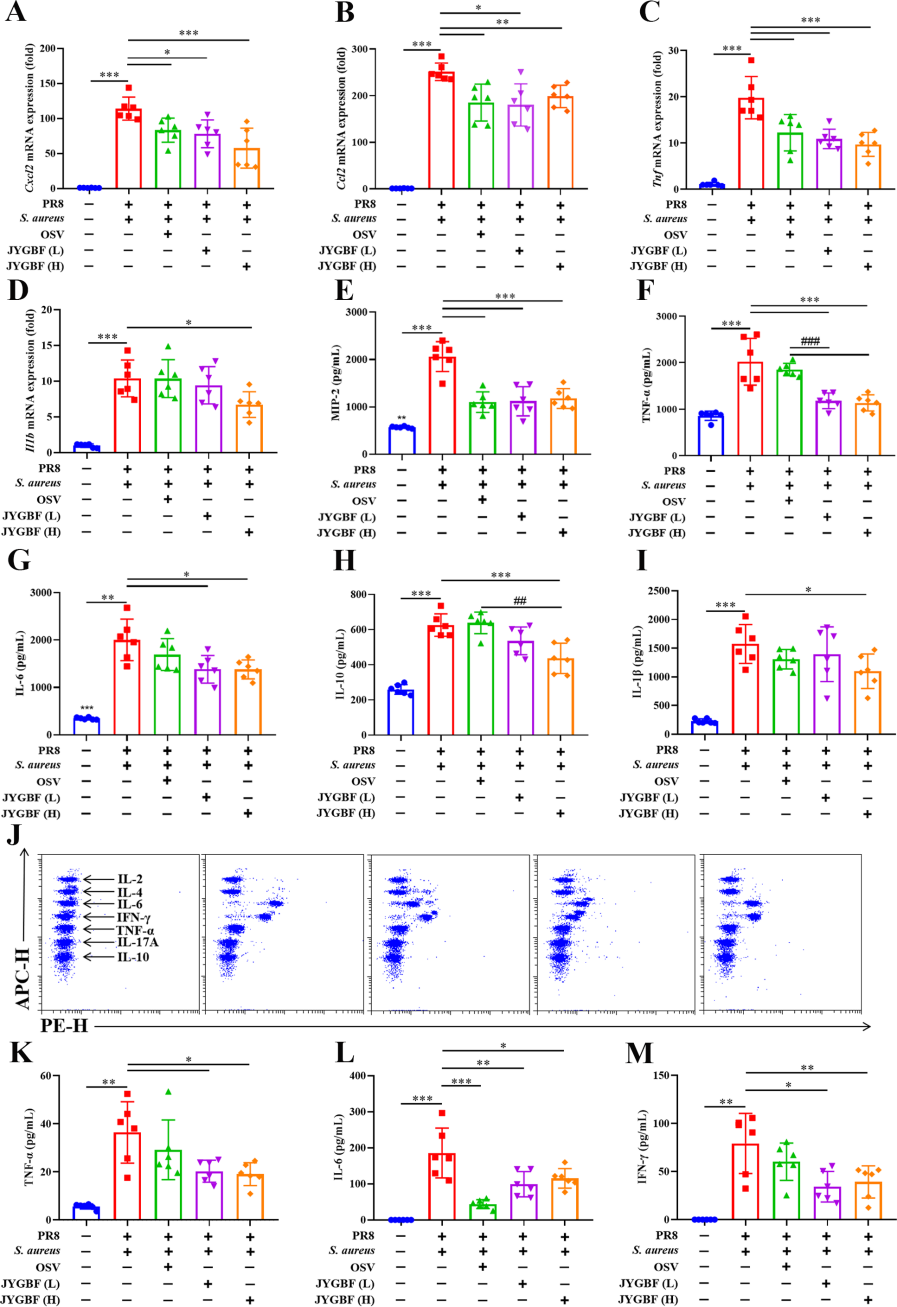
JYGBF inhibited the excessive inflammatory response in postinfluenza *S. aureus*-infected mice. **(A–D)** The mRNA expression of *Cxcl2*, *Ccl2*, *Tnf* and *Il1b* in lungs at 6 dpi (n=6). **(E–I)** The secretion of MIP-2, TNF-α, IL-6, IL-10 and IL-1β in lungs at 6 dpi (n=6). **(J)** The typical diagram of CBA assays at 6 dpi (n=6). **(K–M)** The protein expression of IL-6, TNF-α and INF-γ in sera at 6 dpi (n=6). Data are shown as mean ± SD. *, *p* < 0.05; **, *p* < 0.01; ***, *p* < 0.001; *vs*. PR8 + *S. aureus* group. ##, *p* < 0.01; ###, *p* < 0.001; *vs*. PR8 + *S. aureus* + OSV group.

Secondary bacterial infection could lead to massive production of cytokines and chemokines that recruit inflammatory cells to accelerate severe immunopathology during flu. An excessive expression of inflammatory mediators (*Ccl4*, *Cxcl10*, *Ccl2*, *Cxcl1*, *Cxcl2*, *Ccl7*, *Cxcl13*, *Ifng* and *Il6*) was observed in postinfluenza *S. aureus* infection group compared with those in PR8 group at 6 dpi (**Supplementary Figures S2**). Elevated expression of chemokines during secondary infection might account for the increased infiltration of neutrophils and inflammatory monocytes in lungs (**Supplementary Figures S2**). The mRNA levels of *Cxcl2*, *Ccl2*, *Tnf* and *Il1b* were significantly up-regulated in the lungs of mice during postinfluenza *S. aureus* infection group (**Figures 3A–D**). JYGBF treatment could significantly decrease the mRNA expression of *Cxcl2*, *Ccl2*, *Tnf* and *Il1b* (**Figures 3A–D**). Additionally, the expression levels of MIP-2, TNF-α, IL-6, IL-10 and IL-1β in lung homogenates were also significantly increased in postinfluenza *S. aureus* infection group, while they were significantly down-regulated by JYGBF (**Figures 3E–I**).

In addition, the expression of IL-6, TNF-α and IFN-γ were also significantly increased in sera of postinfluenza *S. aureus* infection group (**Figures 3J–M**). Different doses of JYGBF significantly suppressed IL-6, TNF-α and IFN-γ secretion in sera (**Figures 3J–M**). OSV treatment also decreased the expression of IL-6 in sera (**Figure 3L**). These results indicated that JYGBF treatment could alleviate the overactivated inflammatory response during postinfluenza *S. aureus* infection.

### 3.4 JYGBF alters the proportions of immune cell subsets and inhibits the cytokine score in lungs using scRNA-seq technique

**Figure 4:**
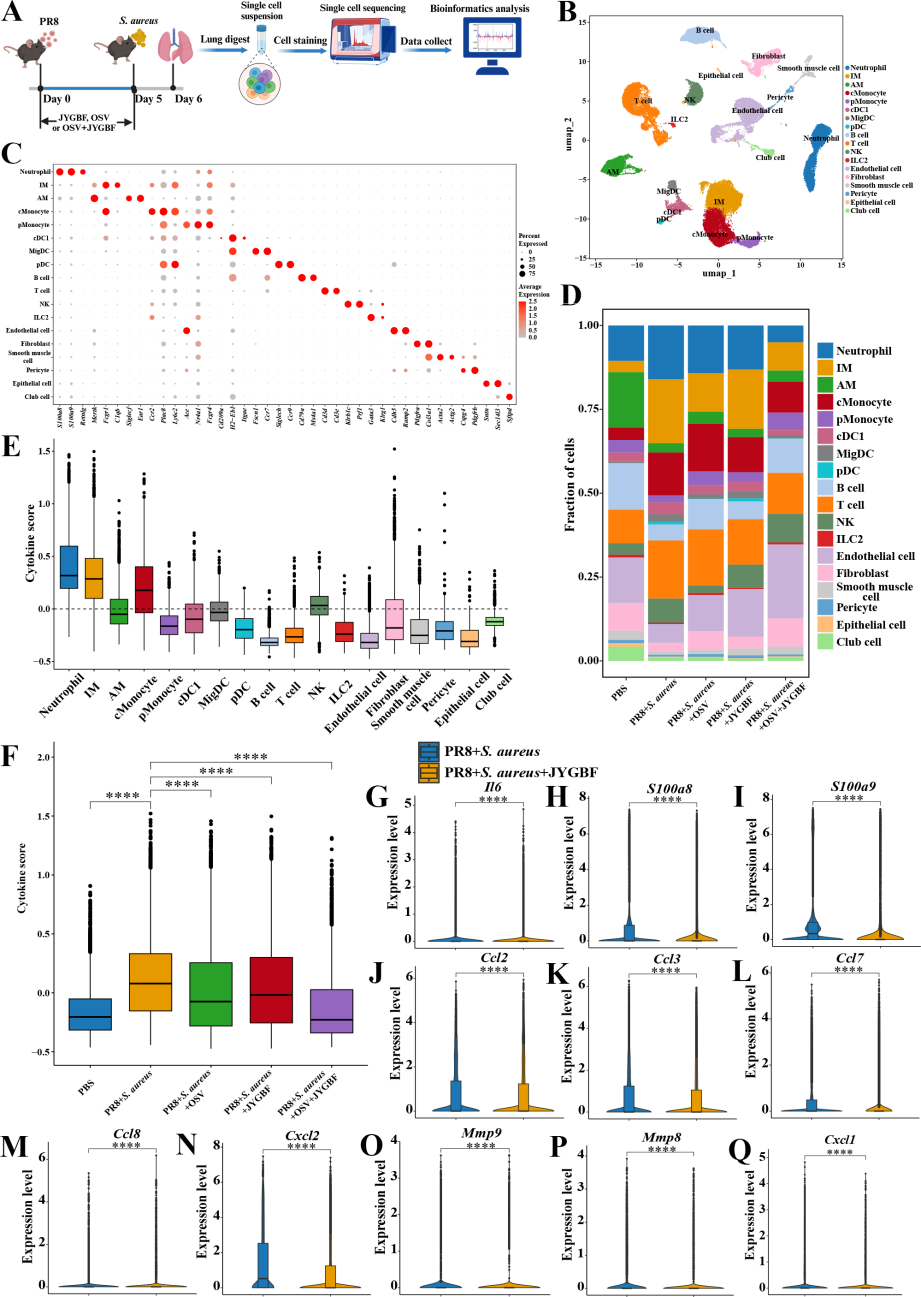
JYGBF altered the proportions of immune cells and reduced the cytokine scores in lungs using scRNA-seq technique. **(A)** Study flow chart of scRNA-seq. **(B)** 18 cell types in lung visualized by UMAP, including neutrophil, interstitial macrophage (IM), alveolar macrophage (AM), classical monocyte (cMonocyte), patrolling monocyte (pMonocyte), type 1 conventional dendritic cells (cDC1), migratory dendritic cell (migDC), plasmacytoid DC (pDC), B cell, T cell, NK, ILC2, Endothelial cell, Fibroblast, Smooth muscle cell, Pericyte, Epithelial cell, Club cell. **(C)** Classical markers were used to annotate 18 cell types. **(D)** The proportions of 18 cell types in 5 groups. **(E)** The cytokine scores of 18 cell types. **(F)** The total cytokine score of lungs in 5 groups. **(G–Q)** JYGBF inhibited the expression of *Il6*, *S100a8*/*a9*, *Ccl2*, *Ccl3*, *Ccl7*, *Ccl8*, *Cxcl2*, *Mmp9*, *Mmp8* and *Cxcl1* in lungs. ****, *p* < 0.0001; vs. PR8 + *S. aureus* group.

To explore the ratio and functions of immune cells influenced by JYGBF treatment during postinfluenza *S. aureus* infection, scRNA-seq was employed to decipher the underlying mechanism (**Figure 4A**). A total of 55,235 cells were obtained from 5 groups including PBS group, PR8 + *S. aureus* group, PR8 + *S. aureus* + OSV group, PR8 + *S. aureus* + JYGBF group and PR8 + *S. aureus* + OSV + JYGBF group. A total of 26 cell clusters were identified as 18 cell types using classic markers (**Figures 4B, C**).

ScRNA-seq data showed an increase in the ratios of neutrophil, IM, cMonocyte, T cell and NK, and a decrease in the ratios of AM, B cell, pMonocyte, endothelial cell, fibroblast, epithelial cell and club cell were observed in lungs of postinfluenza *S. aureus* infection compared with PBS group (**Figure 4D**). JYGBF reduced the ratio of neutrophil, cMonocyte and T cell, and reversed the reduction of endothelial cell (**Figure 4D**). JYGBF did not reduce the ratio of IM in lungs (**Figure 4D**). Neutrophil and IM exhibited the highest cytokine score among these subtypes (**Figure 4E**), which revealed that they might play an important role in the formation of cytokine storm. Furthermore, JYGBF reduced the cytokine score and the expression of inflammatory mediators (*Il6*, *S100a8/a9*, *Ccl2*, *Ccl3*, *Ccl7*, *Ccl8*, *Cxcl2*, *Mmp8, Mmp9* and *Cxcl1*) in lungs (**Figures 4F–Q**). The inhibition of neutrophil infiltration and the downregulation of cytokine score might contribute to the anti-inflammatory role of JYGBF.

### 3.5 JYGBF reduces neutrophil chemotaxis in lungs

**Figure 5:**
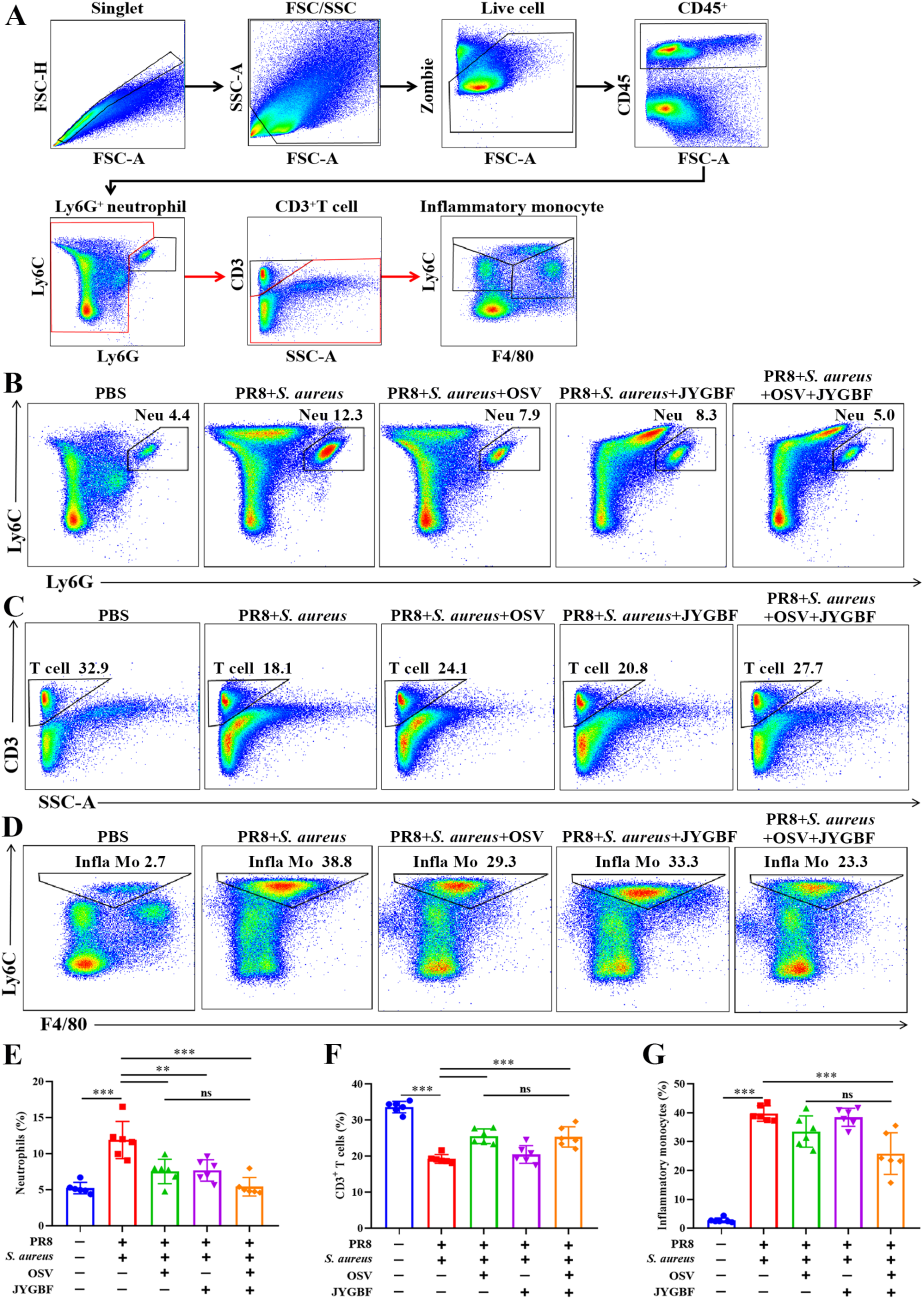
Impacts of JYGBF on immune cells in lungs at 6 dpi. **(A)** The analytical strategy of immune cells in lungs by Flow cytometry. Myeloid cells (CD45^+^ cells), neutrophils (CD45^+^ Ly6G^+^ cells), T cells (CD45^+^ Ly6G^-^ CD3^+^ cells) and inflammatory monocytes (CD45^+^ Ly6G^-^ CD3^-^ Ly6C^+ to hi^ F4/80^- to mid^). **(B–D)** Typical diagrams of neutrophils, T cells and inflammatory monocytes in each group. **(E–G)** The ratios of neutrophils, T cells and inflammatory monocytes in lung tissues at 6 dpi (n=6). Data are shown as mean ± SD. **, *p* < 0.01; ***, *p* < 0.001; *vs*. PR8 + *S. aureus* group. ns, not significant; *vs*. PR8 + *S. aureus* + OSV group.

To further validate the inhibitory effects of JYGBF on the chemotaxis of neutrophils and monocytes, Flow cytometry was used to analyze the immune cells in lungs. Secondary *S. aureus* infection following PR8 infection caused a significant increase in the proportions of neutrophils and inflammatory monocytes compared with PBS group (**Figures 5A–G**). Meanwhile, a decrease in the proportions of T cells was observed in postinfluenza *S. aureus* infection group compared with PBS group (**Figure 5F**). Consistent with the scRNA-seq data (**Figure 4D**), JYGBF, OSV and their combined treatment markedly reduced the infiltration of neutrophils in lungs (**Figure 5E**, **Supplementary Figure S3A**). Inflammatory monocytes from peripheral blood can usually differentiate to IMs, contributing to overactivated inflammatory response (35). Though JYGBF could not decrease the proportions of inflammatory monocytes, the combined treatment significantly reduced the infiltration of inflammatory monocytes in lungs (**Figure 5G**, **Supplementary Figure S3C**). These results indicated that JYGBF could reduce the infiltration of inflammatory innate immune cells in lungs, which is beneficial to alleviate immunopathologic damage caused by overwhelmed inflammatory reaction during secondary infection.

### 3.6 JYGBF inhibits neutrophil activation in lungs

**Figure 6:**
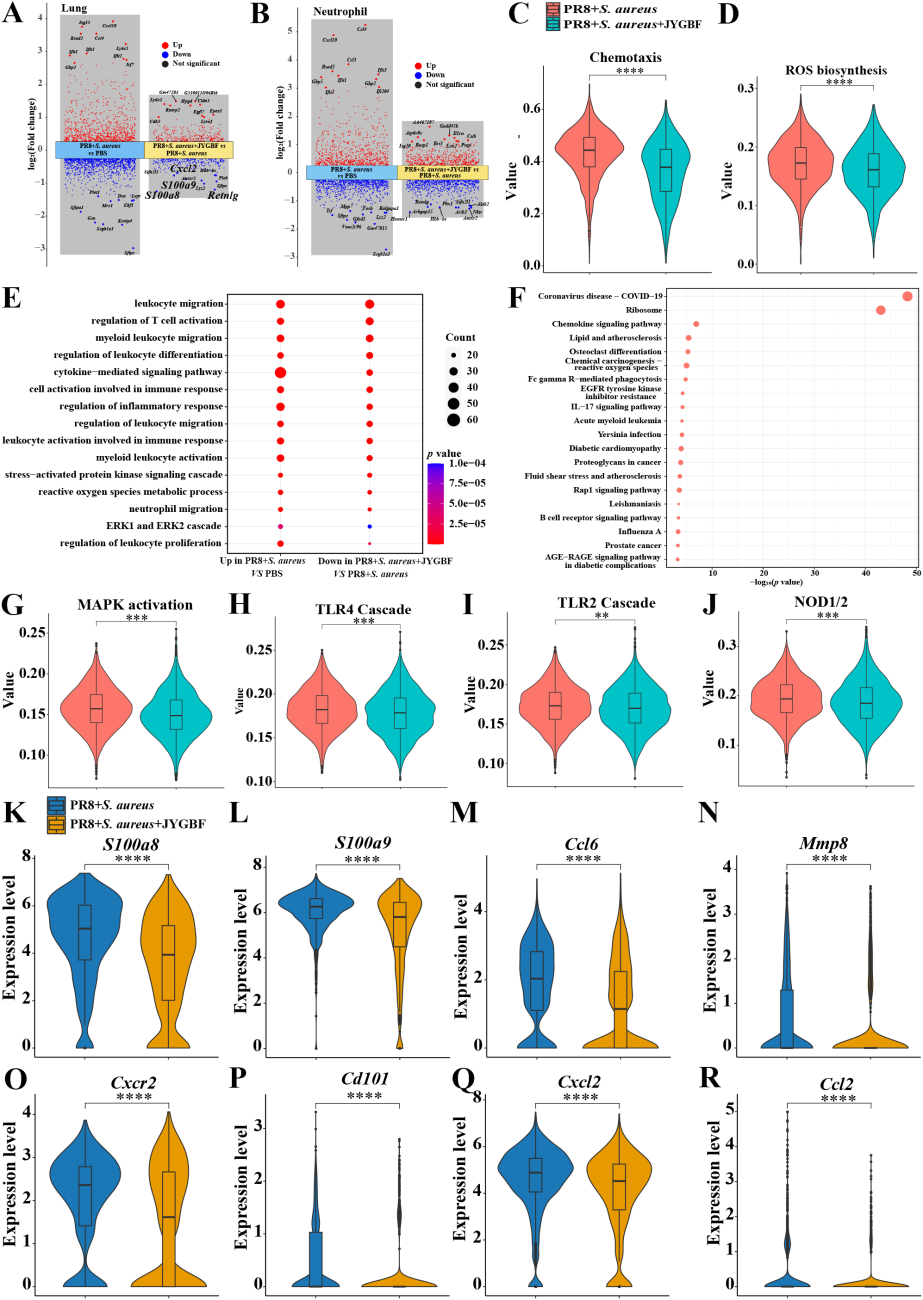
JYGBF inhibited neutrophil activation in lungs. **(A)** Upregulated genes and downregulated genes of neutrophils in postinfluenza *S. aureus* infection group and JYGBF treatment group. **(B)** Upregulated genes and downregulated genes of lung in postinfluenza *S. aureus* infection group and JYGBF treatment group. **(C, D)** JYGBF treatment inhibited neutrophil chemotaxis and ROS biosynthesis. **(E)** GO analysis of downregulated genes in JYGBF treatment group. **(F)** KEGG analysis of downregulated genes in JYGBF treatment group. **(G–J)** GSVA results of MAPK activation, TLR2 cascade, TLR4 cascade and NOD1/2 signaling pathway in neutrophils. **(K–R)** The relative expression of *S100a8/a9, Ccl6, Mmp8, Cxcr2, Cd101, Cxcl2* and *Ccl2* in neutrophils. *p* < 0.05; **p < 0.01; ***p < 0.001; ****p < 0.0001; *vs*. PR8 + *S. aureus* group.

There were 281 up-regulated genes and 249 down-regulated genes in lungs of JYGBF treatment group compared with postinfluenza *S. aureus* infection group (**Figure 6A**). *S100a8/a9*, *Cxcl2* and *Retnlg* were 4 genes in top 10 genes downregulated by JYGBF among them. *S100a8/a9* and *Retnlg* are classic markers of neutrophils. *Cxcl2* is a typical chemokine that can recruit neutrophils. To further explore the regulatory roles of JYGBF on neutrophils, differential gene expression was analyzed in neutrophil population. JYGBF treatment upregulated 664 genes and downregulated 501 genes in neutrophils compared with postinfluenza *S. aureus* infection group (**Figure 6B**). GO terms related to neutrophil activation were used to analyze the effects of JYGBF on neutrophil function. Results indicated that JYGBF could inhibit neutrophil chemotaxis and ROS biosynthesis (**Figures 6C, D**). The downregulated genes regulated by JYGBF were involved in inflammatory response including leukocyte chemotaxis, leukocyte migration, ERK1/2 cascade, etc. (**Figure 6E**). KEGG analysis indicated that the downregulated genes are involved in COVID-19, chemokine signaling pathway, chemical carcinogenesis-reactive oxygen species, influenza, etc. (**Figure 6F**). Consistent with the results from GO analysis, GSVA data also showed that JYGBF could downregulate inflammatory signaling pathways such as MAPK activation, TLR4 cascade, TLR2 cascade, and NOD1/2 signaling pathway (**Figures 6G–J**). Furthermore, JYGBF treatment significantly decreased the expression of neutrophil activation markers and cytokines (*S100a8/a9, Cxcr2, Cd101, Cxcl2, Ccl2, Ccl6* and *Mmp8*) in neutrophils (**Figures 6K–R**). The inhibitory effects on these signaling pathways and overactivated inflammatory response by JYGBF were also seen in IMs (**Supplementary Figure S4**). These results indicated that JYGBF could inhibit the activation of neutrophil and neutrophil-mediated inflammatory response in lungs in postinfluenza *S. aureus* infection mice.

### 3.7 JYGBF inhibits the formation of NETs in lungs

**Figure 7:**
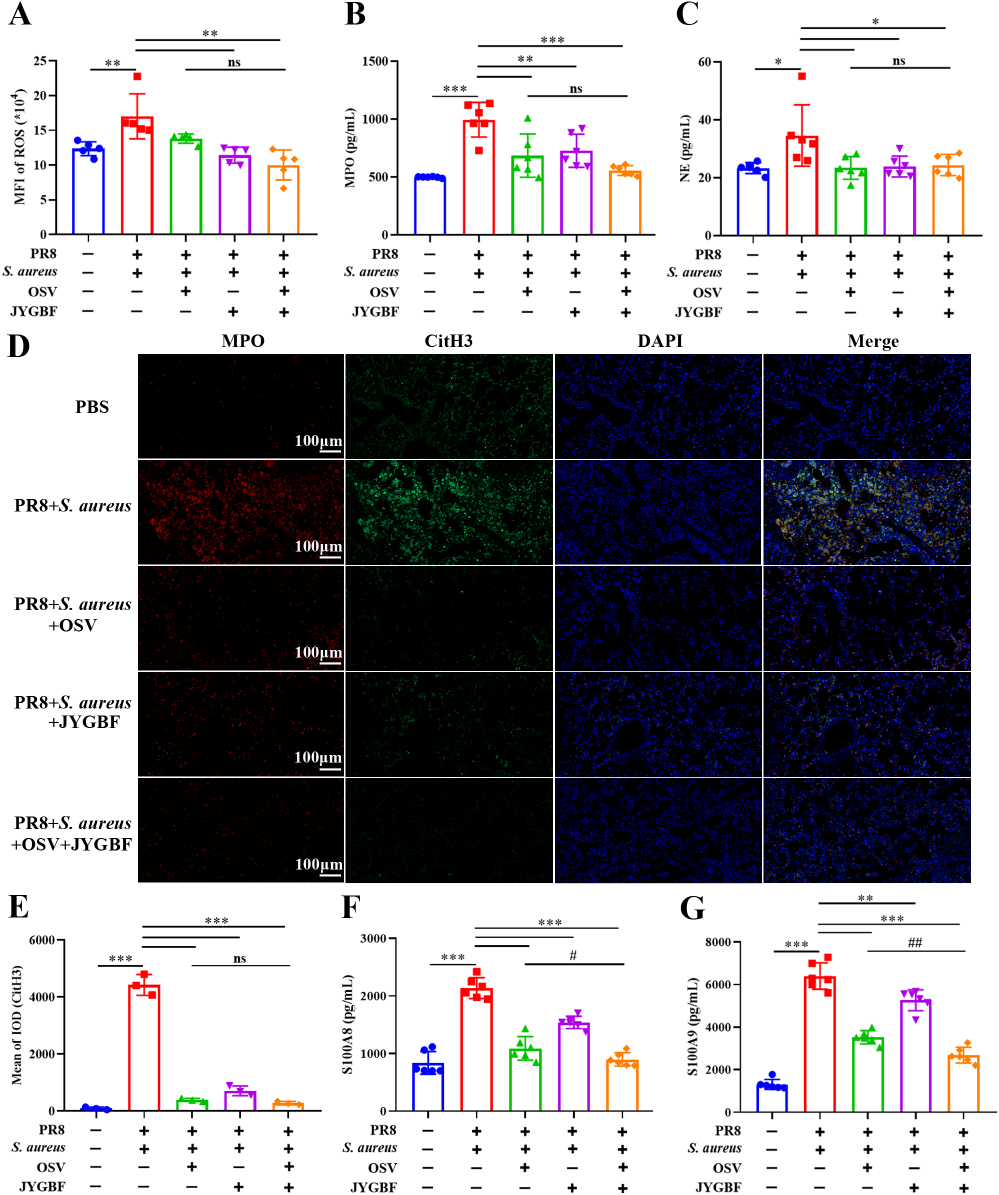
JYGBF inhibited the formation of NETs in lungs. **(A–C)** The production of ROS, MPO and NE in lung tissues (n=5~6) at 6 dpi. **(D)** The expression of MPO, CitH3 and MPO-CitH3 colocalization analyzed by immunofluorescence assays **(E)** The relative expression of CitH3 analyzed by Image-Pro Plus 6.0. **(F, G)** The production of S100A8/A9 in lungs (n=6) at 6 dpi. Data are shown as mean ± SD. *, *p* < 0.05; **, *p* < 0.01; ***, *p* < 0.001; *vs*. PR8 + *S. aureus* group. ns, not significant; #, *p* < 0.05; ##, *p* < 0.01; *vs*. PR8 + *S. aureus* + OSV group.

Activated neutrophils could contribute to the formation of NETs. The release of NETs is a weapon to restrict and eliminate pathogens but also results in lung damage (14). This study has found that JYGBF could decrease the infiltration of neutrophils and inhibit neutrophil activation. In the postinfluenza *S. aureus* infection group, the lungs of mice exhibited an increased level of ROS, MPO and NE compared with those in PBS group (**Figures 7A–C**). JYGBF or the combination of OSV and JYGBF significantly decreased the production of ROS, MPO and NE (**Figures 7A–C**). The colocalization of citrullinated histone H3 (CitH3) and MPO is a well-known marker of NETs. Significantly increased expression of MPO, CitH3 and MPO-CitH3 complexes was observed in lungs of postinfluenza *S. aureus* infection group (**Figures 7D, E**). JYGBF, OSV and the combination of OSV and JYGBF significantly decreased the expression of MPO, CitH3 and MPO-CitH3 complexes in lungs (**Figures 7D, E**). S100A8/A9 can be released by means of NETs and contribute to platelet activation. JYGBF, OSV and their combined treatment could reduce the increased expression of S100A8/A9 in lungs (**Figures 7F, G**). Interestingly, the combination of OSV and JYGBF was superior to OSV in reducing the expression of S100A8/A9 in lungs (**Figures 7F, G**).

**Figure 8:**
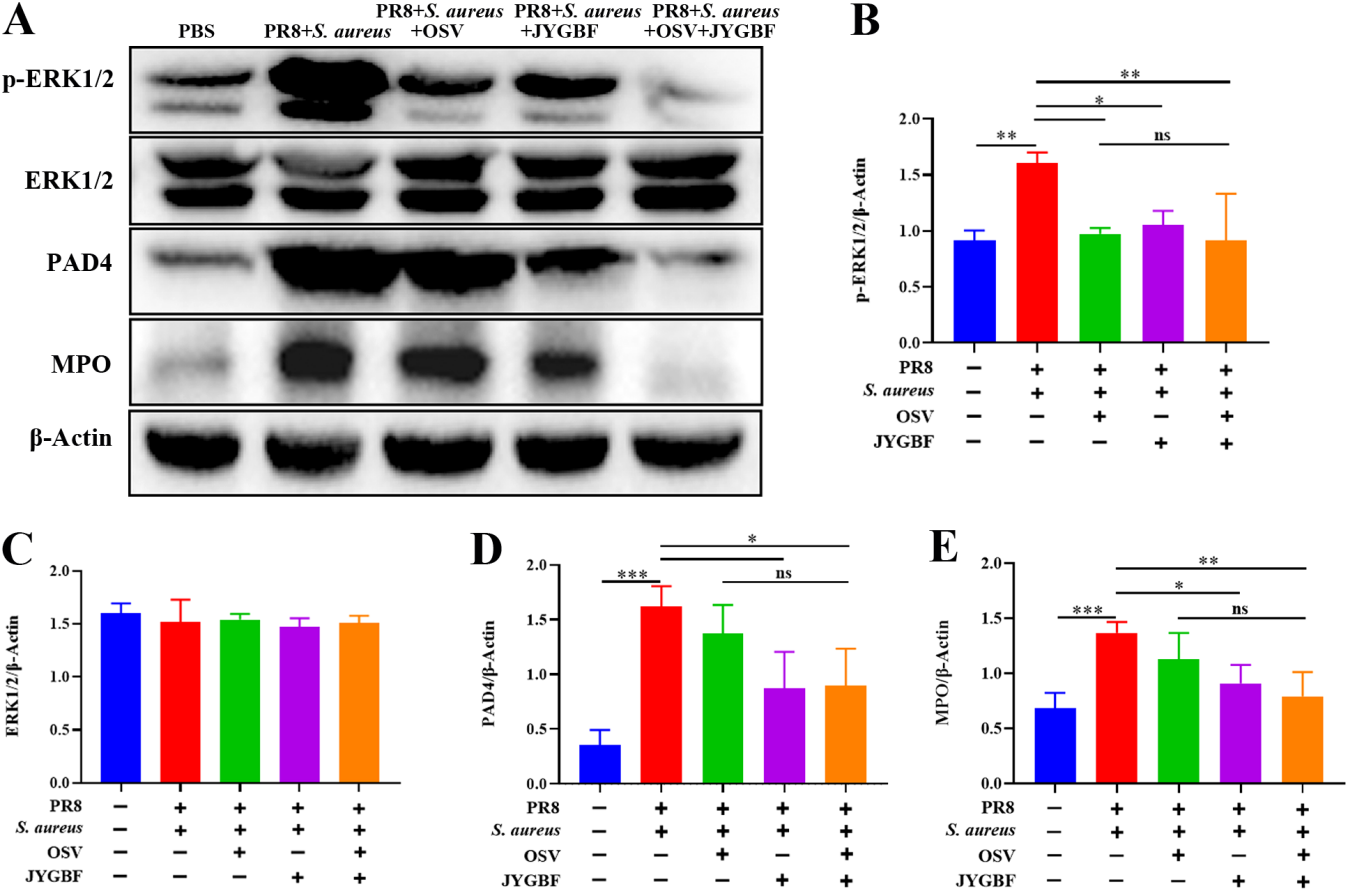
JYGBF inhibited the formation of NETs by ERK/ROS axis. **(A)** Immunoblot analysis of phospho-ERK1/2, ERK1/2, PAD4, MPO and β-Actin (n=3). **(B–E)** The relative expression of phospho-ERK1/2, ERK1/2, MPO and PAD4 analyzed by Image J. Data are shown as mean ± SD. *, *p* < 0.05; **, *p* < 0.01; ***, *p* < 0.001; vs. PR8 + *S. aureus* group. ns, not significant; vs. PR8 + *S. aureus* + OSV group.

Furthermore, the phosphorylation level of ERK1/2 and the expression of PAD4 and MPO significantly increased in lungs of postinfluenza *S. aureus* infection group (**Figures 8A–E**). JYGBF, OSV and the combination of OSV and JYGBF significantly reduced the phosphorylation level of ERK1/2 (**Figures 8A–E**). JYGBF and combined treatment markedly decreased the expression of PAD4 and MPO (**Figures 8A–E**). These findings indicated that JYGBF could inhibit the formation of NETs probably by suppressing the ERK/ROS axis.

### 3.8 JYGBF decreases platelet activation probably by inhibiting NETs in secondary infected mice

**Figure 9:**
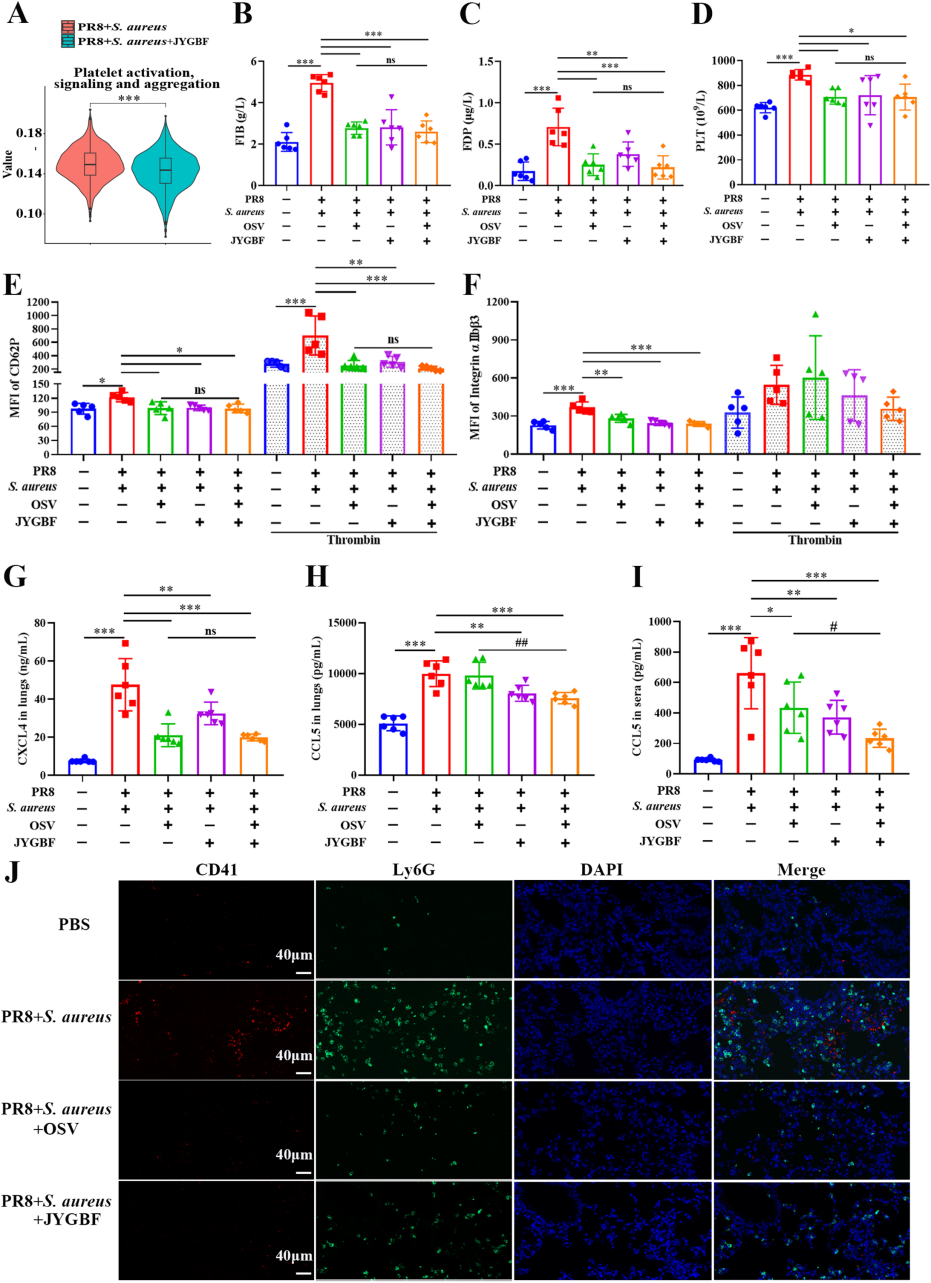
JYGBF inhibited the activation of platelets in secondary infection mice. **(A)** GSVA data of platelet activation, signaling and aggregation. **(B, C)** The production of FIB and FDP in the sera of mice at 7 dpi (n=6). **(D)** The platelet counts in sera at 7 dpi (n=6). **(E, F)** The fluorescence intensity of CD62P and αIIbβ3 in PRP with or without the stimulation of thrombin at 7 dpi (n=5). **(G, H)** The expression of CXCL4 and CCL5 in lungs (n=6). **(I)** The expression of CCL5 in sera (n=6). **(J)** The expression of CD41, Ly6G and platelet-neutrophil aggregates by immunofluorescence assays at 7 dpi. Data are mean ± SD for each group. *, *p* < 0.05; **, *p* < 0.01; ***, *p* < 0.001; *vs*. PR8 + *S. aureus* group. ns, not significant; #, *p* < 0.05; ##, *p* < 0.01; vs. PR8 + *S. aureus* + OSV group.

The formation of NETs could contribute to activated platelets that augment thrombosis. GSVA data from neutrophils indicated that JYGBF could inhibit platelet-activated signaling including platelet activation, signaling & aggregation (**Figure 9A**, **Supplementary Figure S5**). In postinfluenza *S. aureus* infection group, mice exhibited significantly elevated levels of FIB & FDP, and increased platelet counts in sera at 7 dpi. FIB and FDP are typical markers of hypercoagulable status. JYGBF, OSV and the combination of OSV and JYGBF significantly reduced the production of FIB & FDP, and platelet counts (**Figures 9B–D**).

Activated platelets could increase the expression of CD62P, αIIbβ3 and chemokines such as CXCL4 and CCL5. In the postinfluenza *S. aureus* infection group, increased platelet counts and elevated level of platelet activation markers (CD62P, αIIbβ3, CXCL4 and CCL5) were observed in sera or lungs (**Figures 9E–I**). JYGBF, OSV and the combination of OSV and JYGBF could significantly reduce the expression of CD62P, αIIbβ3, CXCL4 and CCL5 in sera or lungs (**Figures 9E–I**). Compared with PBS group, an increased expression of CD62P in PRP stimulated with thrombin was observed in postinfluenza *S. aureus* infection group (**Figure 9E**), which indicated that platelets of postinfluenza *S. aureus* infection group exhibited hyper-reactive status. Reduced expression of CD62P from PRP exposed to thrombin was observed in JYGBF, OSV and combined treatment group (**Figure 9E**). OSV treatment alone did not inhibit the expression of CCL5 in lungs and only slightly reduced CCL5 secretion in sera (**Figures 9H, I**). CD41 and Ly6G are markers of platelets and neutrophils, respectively. Activated platelets interact with neutrophils and drive the formation of platelet-neutrophil aggregates. JYGBF and OSV treatment could significantly reduce the formation of platelet-neutrophil aggregates (**Figure 9J**). Surprisingly, JYGBF could not inhibit platelet aggregation induced by ADP *in vitro* (**Supplementary Figures S6**). These results indicated that JYGBF treatment could inhibit platelet activation in infected mice probably by inhibiting NETs.

### 3.9 JYGBF inhibited the overactivated inflammatory response by ERK MAPK pathway in neutrophils

**Figure 10:**
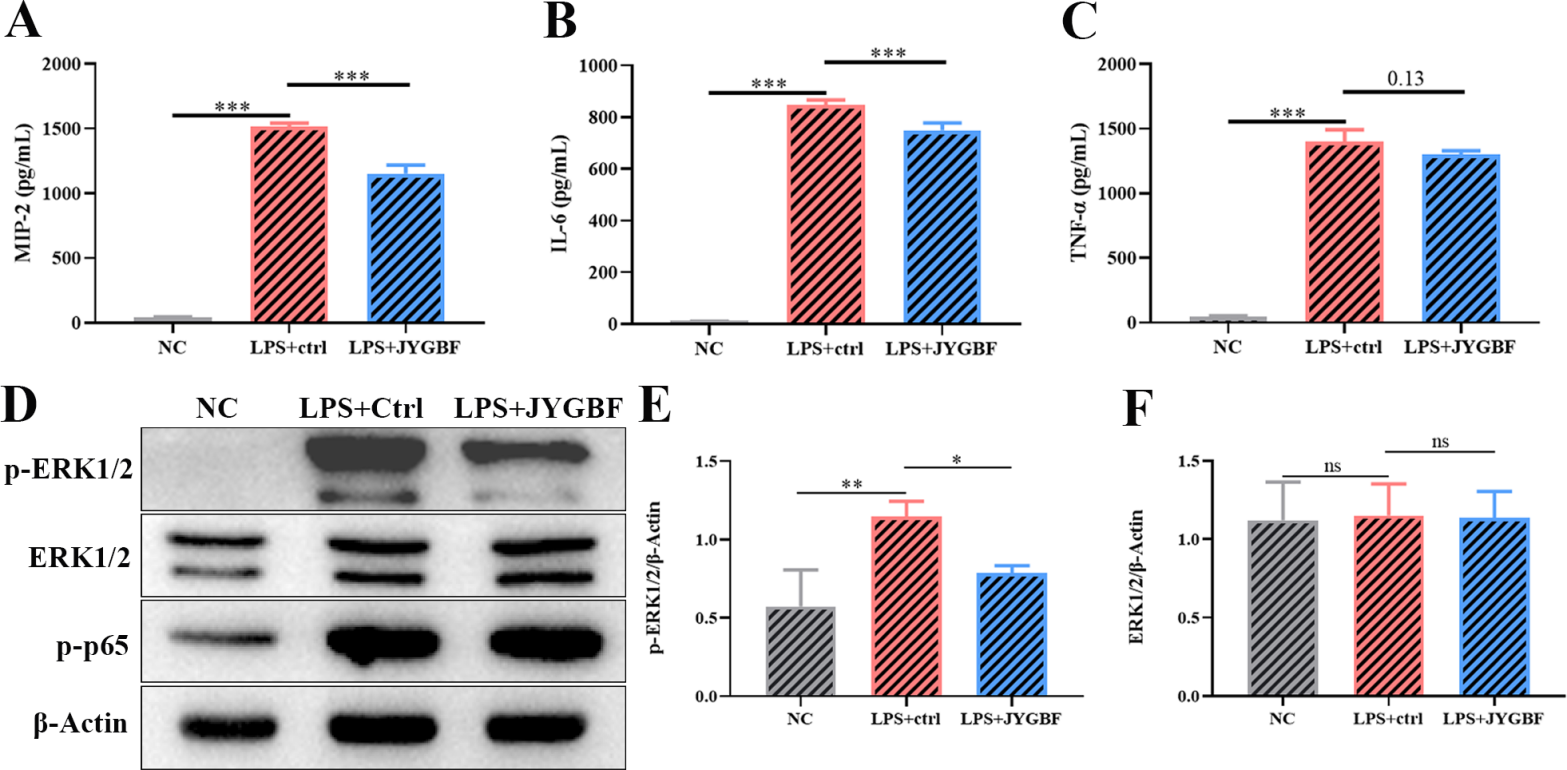
JYGBF inhibits overactivated inflammatory response in neutrophils stimulated with LPS. **(A-C)** The expression of MIP-2, IL-6 and TNF-α in neutrophils stimulated with LPS with the treatment of control sera (ctrl) or JYGBF-containing sera (JYGBF) (n=4). **(D)** The expression of β-Actin, phosphorylated ERK1/2, ERK1/2 and phosphorylated p65 in neutrophils stimulated with LPS (n=3). **(E, F)** The relative expression of phospho-ERK1/2 and ERK1/2 analyzed by Image J. Data are shown as mean ± SD. ns, not significant; *, *p* < 0.05; **, *p* < 0.01; ***, *p* < 0.001; vs. LPS + ctrl group.

Pathogen-associated molecular patterns (PAMPs) are typically activated pattern recognition receptors in viral or bacterial infections. Lipopolysaccharide (LPS) is a common PAMP of Gram-negative bacteria, which could be derived from bacterial translocation for impaired intestinal integrity (36). To validate the regulatory role of JYGBF on ERK/ROS axis, LPS was used to activate ERK MAPK pathway. The purity of CD11b^+^Ly6G^+^ neutrophils were determined by FACS and the neutrophils were stimulated by LPS (**Supplementary Figure S7**). LPS stimulation could induce increased expression of MIP-2, IL-6 and TNF-α in neutrophils, while JYGBF decreased the expression of MIP-2 and IL-6 (**Figures 10A–C**). Furthermore, LPS stimulation induced the high phosphorylation of ERK1/2 (**Figures 10D–F**). JYGBF could reduce ERK phosphorylation (**Figures 10D–F**), which is consistent with the downregulating role on ERK activation *in vivo* by JYGBF.

### 3.10 The active component of JYGBF, acacetin, inhibited the formation of NETs via ERK/ROS axis

**Figure 11:**
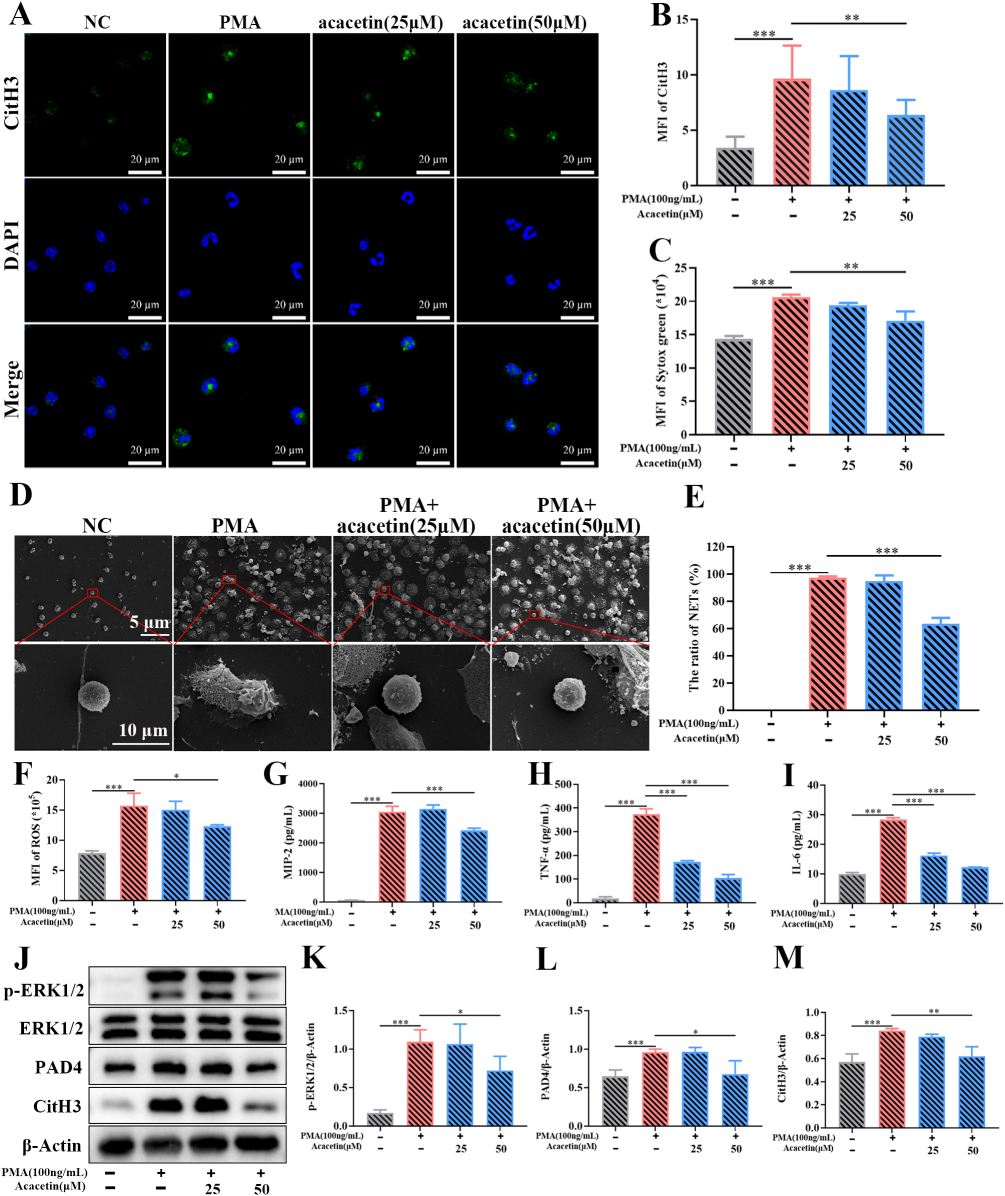
Acacetin inhibits the PMA-induced NETs via inhibiting ERK/ROS axis. **(A)** The expression of CitH3 assessed by immunofluorescence assays (n=3). **(B)** The relative expression of CitH3 analyzed by ZEN (n=3). **(C)** MFI of Sytox green (n=3). **(D, E)** The formation of NETs examined by scanning electron microscopy (n=3). **(F–I)** The production of ROS, MIP-2, TNF-α and IL-6 in neutrophils stimulated by PMA (n=3). **(J)** The expression of phosphorylated ERK1/2, ERK1/2, PAD4, CitH3 and β-Actin in neutrophils stimulated by PMA (n=3). **(K–M)** The relative expression of phospho-ERK1/2, PAD4 and CitH3 analyzed by Image J. Data are shown as mean ± SD. *p < 0.05; **p < 0.01; ***p < 0.001; *vs*. PMA group.

A total of 30 compounds were identified in the sera of mice after 7 consecutive days’ gavage of JYGBF (**Supplementary Figure S8**, **Supplementary Table 3**). Acacetin is a critical component of JYGBF (**Supplementary Table 3**). PMA is a PKC agonist and could induce the formation of NETs rapidly by PKC/ERK/ROS axis. Neutrophils were stimulated by PMA and treated with acacetin simultaneously. PMA stimulation led to increased expression and release of CitH3 (**Figures 11A, B**). As shown in **Figures 11A, B**, acacetin inhibited the increase of CitH3. Sytox green, a DNA binding-dye, was used to stain dead cells and to assess the formation of NETs. As shown in **Figure 11C**, acacetin reduced the formation of NETs induced by PMA. Consistently, the inhibitory effects of JYGBF on NETosis were determined by scanning electron microscopy (**Figures 11D, E**). PMA stimulation could induce the production of ROS, MIP-2, TNF-α and IL-6 in neutrophils, while acacetin inhibited the increased production of ROS, MIP-2, TNF-α and IL-6 (**Figures 11F–I**). Besides, PMA stimulation increased the phosphorylation level of ERK1/2, and the expression levels of PAD4 and CitH3 in neutrophils. But they were also reduced by acacetin (**Figures 11J–M**). These results indicated that acacetin inhibited the activation of ERK/ROS axis, thus downregulated PAD4 and subsequent CitH3 expression in neutrophils.

## 4 Discussion

Bacterial coinfection is one of the main causes of severe cases in patients of influenza or COVID-19, which is characterized by overwhelmed inflammatory response and hypercoagulation (13, 37–41). JYGBF has been proven to benefit COVID-19 patients in a clinical study enrolled 950 patients (31). This study demonstrates that JYGBF could also protect mice from postinfluenza *S. aureus* infection including the increased survival rate of infected mice, symptom relief and body temperature maintenance. Neutrophils, inflammatory monocytes and IMs are the main contributors to the secretion of chemokines and cytokines at the early stage of infection, which results in immunopathological damage (42). During infection, inflammatory monocytes usually undergo differentiation into interstitial macrophages that could produce high levels of matrix metallopeptidases and pro-inflammatory cytokines to exacerbate lung damage (35). Massive neutrophil infiltration could damage the epithelial-endothelial barrier by releasing ROS and proteases (12, 14). Neutrophil-secreted mediators such as S100A8/A9 and chemokines could lead to a more aggressive inflammatory response, and recruit more immune cells into the lungs aggregating the severity of the disease (43). S100A8/A9 are known to be the key drivers of cytokine storm (42). Our data from scRNA-seq indicate that neutrophils showed the highest cytokine score followed by IMs among the 18 cell clusters in lungs. A recent study found that innate immune cells especially neutrophils exhibit significantly higher cytokine scores in severe bacterial patients, which are thought to be the predominant drivers of overactivated inflammatory response and contributed significantly to the pathogenesis of severe disease (42). *In vivo* studies also revealed that increased neutrophil infiltration and neutrophil-mediated immune response are observed in the current secondary infected model, which is similar to postinfluenza *Pseudomonas aeruginosa* infection (11). Thereby, neutrophils played an important role in the pathogenesis of postinfluenza *S. aureus* infection. Notably, JYGBF could reduce neutrophil infiltration and the activation of neutrophils and IMs. This study indicates that controlling neutrophil chemotaxis and neutrophil-mediated inflammatory response is one of the mechanisms that JYGBF mitigates ALI in infected mice.

Though activated neutrophils capture, trap and kill invasive bacteria in the form of NETs, excessive NET formation also contributes to immunopathological damage during postinfluenza *S. aureus* infection (12). NETosis is also an important driver of the activation of procoagulant platelets and immunothrombosis during postinfluenza bacterial infection or COVID-19 (12, 37, 44). Inhibiting NETosis may be a promising strategy to prevent postinfluenza bacterial infection from mild to severe infection. ROS are central players in the formation of NETosis, which are implicated in initiating chromatin decondensation by increasing the expression of PAD4 and MPO (14). Activated PAD4 citrullinates histones and subsequently results in chromatin decondensation. MPO mediates the activation and translocation of NE which can degrade F-actin filaments and subsequently enter the nucleus to disrupt chromatin packaging. The plasma membrane rupture leads to the release of NET structure including chromatin DNA filaments, histones and granule proteins. The release of ROS, DNA and proteases during NETosis impairs the endothelial barrier, increases endothelial permeability and results in extensive microvascular leakage in lungs (14, 45). NET formation could also result in poor outcomes in patients with respiratory infectious diseases (16, 18). The activation of ERK MAPK signaling pathway is essential for regulating cytokine expression and priming of ROS production through the activation of NADPH oxidase (14, 15). This study reveals that JYGBF can inhibit NETosis via ERK/ROS axis in postinfluenza *S. aureus* infected mice and therefore alleviates ALI.

The formation of NETs not only damages lung tissue but also is an integral contributor to immunothrombosis (44). S100A8/A9 are highly expressed in neutrophils (**Figure 4C**) comprising 40% of the detergent-soluble protein content in the cytoplasm, and can promote platelet activation and clotting via S100A8/A9-GPIbα axis (17, 43). The content of NETs, histones, elicits platelet activation and aggregation in a TLR2/4-dependent manner. All of the extracellular DNA, histones and PAD4 in NETs can interact with von Willebrand Factor, damage the vasculature and further promote thrombus formation (14, 46, 47). The previous factors promote the formation of immuno-thrombosis. The coinfection of influenza virus and bacteria has a higher risk of developing severe coagulopathy such as purpura fulminans and even DIC (38, 39), which is associated with worse outcomes. NETosis is a key driver of pathogenic thrombosis in postinfluenza bacterial infection, severe influenza or COVID-19 (16, 37, 48). FIB is the most abundant coagulation factor in plasma and also an important reaction substrate for thrombosis, which is mainly synthesized by hepatocytes. FDP is produced from fibrinogen breakdown and its excessive production is usually associated with hypercoagulable state or DIC (49). This study indicates that postinfluenza *S. aureus* infection leads to elevated levels of FIB & FDP, and increased platelet counts in mice, which are coherent with a hypercoagulable state coupled with a hyperinflammation state (44). ScRNA-seq data revealed that JYGBF could inhibit neutrophil-platelet interaction and neutrophil-associated platelet activation. *In vivo* studies also confirm that JYGBF improves the hypercoagulable state and inhibits platelet activation probably by inhibiting NETosis. Therefore, JYGBF could inhibit NET-mediated thrombosis and prevent the occurrence of coagulation disorders.

A total of 30 active compounds including acacetin, luteolin and hesperetin are identified in JYGBF-containing sera. Multiple components among 30 active compounds could reduce ROS production. Notably, acacetin can inhibit the formation of NETs and inflammatory response by inhibiting ERK/ROS axis. Luteolin and hesperetin have been shown to inhibit the formation of NETs by inhibiting Raf1/MEK-1/ERK and ROS/autophagy signaling pathways, respectively (50, 51). In a word, acacetin, luteolin and hesperetin could be the main material basis that accounts for the inhibitory effects of JYGBF on NETosis.

Postinfluenza bacterial infection contributes to a high morbidity of severe cases compared with influenza virus infection alone due to the overactivated and dysregulated inflammatory response. Though OSV is a critical antiviral agent and could reduce the release of progeny viruses, it could not directly control the overactivated inflammatory response and is not sufficient to treat patients with secondary bacterial infections. Additionally, OSV treatment exhibits poor efficacy unless it is taken at the early stage during influenza. Chinese medicine could usually benefit patients by inhibiting the overactivated inflammatory response in infectious disease and effectively improve the poor outcomes during influenza or COVID-19 (19, 32, 52). The combination of Chinese medicine with antiviral agents is a promising strategy to treat viral infections, which is recommended in China (53, 54). In this study, JYGBF treatment is superior to OSV in inhibiting the overactivated inflammatory response such as TNF-α, IL-1β and IL-10. In addition, the combination of OSV and JYGBF is superior to OSV in alleviating clinical symptoms and mitigating lung damage caused by secondary infection. The combined treatment has synergistic effects on reducing the infiltration of inflammatory monocytes and decreasing the production of ROS, PAD4, CCL5, S100A8/A9, etc. Therefore, they exhibit synergistic effects on protecting mice from postinfluenza *S. aureus* infection due to their different mechanisms of action.

In conclusion, JYGBF confers a protective effect against postinfluenza *S. aureus* infection. It is mainly attributed to the inhibition of NET formation and overactivated inflammatory response by suppressing the ERK/ROS axis in neutrophils. JYGBF may serve as a promising drug candidate to mitigate the severity of postinfluenza *S. aureus* infection. The combination of OSV and an effective Chinese medicine formula (e.g. JYGBF) might be a promising strategy for the prevention and treatment of postinfluenza bacterial infection.

## Data Availability

The single cell RNA-sequencing data presented in the study are deposited in the NCBI repository, accession number PRJNA1216737.

## References

[1] WHO. Global Influenza Strategy 2019–2030. (2019). Available online at: https://www.who.int/publications/i/item/9789241515320 (Accessed March 02, 2025).

[2] Liu Y Ling L Wong SH Wang MH Fitzgerald JR Zou X. Outcomes of respiratory viral-bacterial co-infection in adult hospitalized patients. eClinicalMedicine. (2021) 37:100955. doi: 10.1016/j.eclinm.2021.100955 34386745 PMC8343259

[3] CDC. Bacterial coinfections in lung tissue specimens from fatal cases of 2009 pandemic influenza A (H1N1) - United States, May-August 2009. MMWR Morb Mortal Wkly Rep. (2009) 58:1071–4. 19798021

[4] Estenssoro E Ríos FG Apezteguía C Reina R Neira J Ceraso DH. Pandemic 2009 influenza A in Argentina: a study of 337 patients on mechanical ventilation. Am J Respir Crit Care Med. (2010) 182:41–8. doi: 10.1164/201001-0037oc 20203241

[5] Shieh WJ Blau DM Denison AM Deleon-Carnes M Adem P Bhatnagar J. 2009 pandemic influenza A (H1N1): pathology and pathogenesis of 100 fatal cases in the United States. Am J Pathol. (2010) 177:166–75. doi: 10.2353/ajpath.2010.100115 PMC2893660 20508031

[6] Morens DM Taubenberger JK Fauci AS. Predominant role of bacterial pneumonia as a cause of death in pandemic influenza: implications for pandemic influenza preparedness. J Infect Dis. (2008) 198:962–70. doi: 10.1086/591708 PMC2599911 18710327

[7] Arranz H Javier JP Sergio RR Alberto UR Jose AAA Lalueza A. Determinants of poor clinical outcome in patients with influenza pneumonia: A systematic review and metaanalysis. Int J Infect Dis. (2023) 131:173–9. doi: 10.1016/j.ijid.2023.04.003 37030656

[8] Hageman JC Uyeki TM Francis JS Jernigan DB Wheeler JG Bridges CB. Severe community-acquired pneumonia due to Staphylococcus aureus 2003-04 influenza season. Emerg Infect Dis. (2006) 12:894–9. doi: 10.3201/eid1206.051141 PMC3373026 16707043

[9] Ni YN Chen G Sun J Liang BM Liang ZA. The effect of corticosteroids on mortality of patients with influenza pneumonia: a systematic review and meta-analysis. Crit Care. (2019) 23:99. doi: 10.1186/s13054-019-2395-8 30917856 PMC6437920

[10] Gao Y Guyatt G Uyeki TM Liu M Chen Y Zhao Y. Antivirals for treatment of severe influenza: a systematic review and network meta-analysis of randomized controlled trials. Lancet. (2024) 404:753–63. doi: 10.1016/s0140-6736(24)01307-2 PMC11369965 39181595

[11] Jie F Wu X Zhang F Li J Liu Z He Y. Influenza Virus Infection Increases Host Susceptibility To Secondary Infection with Pseudomonas aeruginosa, and This Is Attributed To Neutrophil Dysfunction through Reduced Myeloperoxidase Activity. Microbiol Spectr. (2023) 11:e0365522. doi: 10.1128/spectrum.03655-22 36475755 PMC9927171

[12] Yi T Ding W Hao Y Cen L Li J Shi X. Neutrophil extracellular traps mediate severe lung injury induced by influenza A virus H1N1 in mice coinfected with Staphylococcus aureus. Microbial Pathogenesis. (2022) 166:105558. doi: 10.1016/j.micpath.2022.105558 35487479

[13] Yuki K Koutsogiannaki S. Pattern recognition receptors as therapeutic targets for bacterial, viral and fungal sepsis. Int Immunopharmacol. (2021) 98:107909. doi: 10.1016/j.intimp.2021.107909 34182242 PMC8380728

[14] Zhang H Wang Y Qu M Li W Wu D Cata JP. Neutrophil, neutrophil extracellular traps and endothelial cell dysfunction in sepsis. Clin Transl Med. (2023) 13:e1170. doi: 10.1002/ctm2.1170 36629024 PMC9832433

[15] Hakkim A Fuchs TA Martinez NE Hess S Prinz H Zychlinsky A. Activation of the Raf-MEK-ERK pathway is required for neutrophil extracellular trap formation. Nat Chem Biol. (2011) 7:75–7. doi: 10.1038/nchembio.496 21170021

[16] Middleton EA He XY Denorme F Campbell RA Ng D Salvatore SP. Neutrophil extracellular traps contribute to immunothrombosis in COVID-19 acute respiratory distress syndrome. Blood. (2020) 136:1169–79. doi: 10.1182/blood.2020007008 PMC7472714 32597954

[17] Colicchia M Schrottmaier WC Perrella G Reyat JS Begum J Slater A. S100A8/A9 drives the formation of procoagulant platelets through GPIbα. Blood. (2022) 140:2626–43. doi: 10.1182/blood.2021014966 PMC10653093 36026606

[18] Mao JY Zhang JH Cheng W Chen JW Cui N. Effects of neutrophil extracellular traps in patients with septic coagulopathy and their interaction with autophagy. Front Immunol. (2021) 12:757041. doi: 10.3389/fimmu.2021.757041 34707618 PMC8542927

[19] Ye XL Tian SS Tang CC Jiang XR Liu D Yang GZ. Cytokine storm in acute viral respiratory injury: role of qing-fei-pai-du decoction in inhibiting the infiltration of neutrophils and macrophages through TAK1/IKK/NF-κB pathway. Am J Chin Med. (2023) 51:1153–88. doi: 10.1142/s0192415x23500532 37403214

[20] Song J Zhao J Cai X Qin S Chen Z Huang X. Lianhuaqingwen capsule inhibits non-lethal doses of influenza virus-induced secondary Staphylococcus aureus infection in mice. J Ethnopharmacology. (2022) 298:115653. doi: 10.1016/j.jep.2022.115653 35995276

[21] Zhao J Wang Y Huang X Ma Q Song J Wu X. Liu Shen Wan inhibits influenza virus-induced secondary *Staphylococcus aureus* infection *in vivo* and *in vitro* . J Ethnopharmacol. (2021) 277:114066. doi: 10.1016/j.jep.2021.114066 33766755

[22] Hu K Guan WJ Bi Y Zhang W Li L Zhang B. Efficacy and safety of Lianhua Qingwen capsules, a repurposed Chinese herb, in patients with Coronavirus disease 2019: A multicenter, prospective, randomized controlled trial [Phytomedicine 85 (2021) 153242. Phytomedicine. (2022) 94:153800. doi: 10.1016/j.phymed.2021.153800 34775361 PMC8805149

[23] Du Q Huang W Zhao J Zeng J Zhang W Huang X. Lianhuaqingwen capsule inhibits influenza-induced bacterial adhesion to respiratory epithelial cells through down-regulation of cell adhesion molecules. J Ethnopharmacol. (2021) 280:114128. doi: 10.1016/j.jep.2021.114128 33872750

[24] Chen B Geng P Shen J Liangpunsakul S Lyu H Zhang J. “Traditional Chinese Medicine JingYinGuBiao Formula Therapy Improves the Negative Conversion Rate of SARS-CoV2 in Patients with Mild COVID-19.” Int J Biol Sci. (2022) 18(15):5641–52. doi: 10.7150/ijbs.76699 36263182 PMC9576522

[25] Zhang YJ Liu GJ Zhang H Liu C Chen ZQ Xian JS. Effectiveness of lianhua qingwen granule and jingyin gubiao prescription in omicron BA.2 infection and hospitalization: A real-World study of 56,244 cases in shanghai, China. Chin J Integr Med. (2024) 31(1):11–18. doi: 10.1007/s11655-024-3901-7 38910189

[26] Xie X Gu L Xu W Yu X Yin G Wang J. Integrating anti-influenza virus activity and chemical pattern recognition to explore the quality evaluation method of lonicerae japonicae flos. Molecules. (2022) 27(18):5789. doi: 10.3390/molecules27185789 36144525 PMC9502701

[27] Liang Y Zhang Q Zhang L Wang R Xu X Hu X. Astragalus membranaceus treatment protects raw264.7 cells from influenza virus by regulating G1 phase and the TLR3-mediated signaling pathway. Evid Based Complement Alternat Med. (2019) 2019:2971604. doi: 10.1155/2019/2971604 31975996 PMC6955127

[28] Liu H Xu L Lu E Tang C Zhang H Xu Y. Platycodin D facilitates antiviral immunity through inhibiting cytokine storm via targeting K63-linked TRAF6 ubiquitination. J Leukoc Biol. (2024) 117(2):qiae075. doi: 10.1093/jleuko/qiae075 38518381

[29] Yu Y Zhang Y Wang S Liu W Hao C Wang W. Inhibition effects of patchouli alcohol against influenza a virus through targeting cellular PI3K/Akt and ERK/MAPK signaling pathways. Virol J. (2019) 16:163. doi: 10.1186/s12985-019-1266-x 31870450 PMC6929483

[30] Liang X Huang Y Pan X Hao Y Chen X Jiang H. Erucic acid from Isatis indigotica Fort. suppresses influenza A virus replication and inflammation *in vitro* and *in vivo* through modulation of NF-kappaB and p38 MAPK pathway. J Pharm Anal. (2020) 10:130–46. doi: 10.1016/j.jpha.2019.09.005 PMC7192973 32373385

[31] Chen B Geng P Shen J Liangpunsakul S Lyu H Zhang J. Traditional chinese medicine jingYinGuBiao formula therapy improves the negative conversion rate of SARS-coV2 in patients with mild COVID-19. Int J Biol Sci. (2022) 18:5641–52. doi: 10.7150/ijbs.76699 PMC9576522 36263182

[32] Wu X Xu L Xu G Xu Y Liu H Hu Y. Fei-yan-qing-hua decoction exerts an anti-inflammatory role during influenza by inhibiting the infiltration of macrophages and neutrophils through NF-κB and p38 MAPK pathways. J Ethnopharmacol. (2025) 337:118846. doi: 10.1016/j.jep.2024.118846 39306208

[33] Jiang X Chen Y Liu D Shi T Cheng X He W. Secoeudesma sesquiterpenes lactone A alleviates inflammation and offers adjuvant protection in severe infection of carbapenem-resistant Klebsiella pneumoniae. J Ethnopharmacol. (2020) 252:112605. doi: 10.1016/j.jep.2020.112605 31981749

[34] Koszalka P Subbarao K Baz M. Preclinical and clinical developments for combination treatment of influenza. PloS Pathog. (2022) 18(5):e1010481. doi: 10.1371/journal.ppat.1010481 35551301 PMC9098076

[35] Alon R Sportiello M Kozlovski S Kumar A Reilly EC Zarbock A. Leukocyte trafficking to the lungs and beyond: lessons from influenza for COVID-19. Nat Rev Immunol. (2020) 21:49–64. doi: 10.1038/s41577-020-00470-2 33214719 PMC7675406

[36] Dou B Wu X He Y Xu G Zhang H Huang Q. Fei-Yan-Qing-Hua decoction attenuates influenza virus infection by enhancing host antiviral response through microbiota-derived acetate. Front Pharmacol. (2024) 15:1446749. doi: 10.3389/fphar.2024.1446749 39449967 PMC11499185

[37] Walters KA D’Agnillo F Sheng ZM Kindrachuk J Schwartzman LM Kuestner RE. 1918 pandemic influenza virus and Streptococcus pneumoniae co-infection results in activation of coagulation and widespread pulmonary thrombosis in mice and humans. J Pathol. (2016) 238:85–975. doi: 10.1002/path.4638 26383585 PMC4789761

[38] Nguyen T Kyle UG Jaimon N Tcharmtchi MH Coss-Bu JA Lam F. Coinfection with Staphylococcus aureus increases risk of severe coagulopathy in critically ill children with influenza A (H1N1) virus infection. Crit Care Med. (2012) 40:3246–50. doi: 10.1097/CCM.0b013e318260c7f8 PMC3502679 22971587

[39] Brown M Gerrard J McDougall C MacPhail J Williams O. Purpura fulminans in young women with influenza and co-infections. Lancet. (2024) 403:2290–1. doi: 10.1016/s0140-6736(24)00137-5 38796204

[40] Fan H Zhou L Lv J Yang S Chen G Liu X. Bacterial coinfections contribute to severe COVID-19 in winter. Cell Res. (2023) 33:562–4. doi: 10.1038/s41422-023-00821-3 PMC10204009 37221267

[41] To KK Hung IF Li IW Lee KL Koo CK Yan WW. Delayed clearance of viral load and marked cytokine activation in severe cases of pandemic H1N1 2009 influenza virus infection. Clin Infect Dis. (2010) 50:850–9. doi: 10.1086/650581 PMC7107930 20136415

[42] Xiao K Cao Y Han Z Zhang Y Luu LDW Chen L. A pan-immune panorama of bacterial pneumonia revealed by a large-scale single-cell transcriptome atlas. Signal Transduct Target Ther. (2025) 10:5. doi: 10.1038/s41392-024-02093-8 39757231 PMC11701081

[43] Pruenster M Vogl T Roth J Sperandio M. S100A8/A9: From basic science to clinical application. Pharmacol Ther. (2016) 167:120–31. doi: 10.1016/j.pharmthera.2016.07.015 27492899

[44] Bonaventura A Vecchié A Dagna L Martinod K Dixon DL Van TB. Endothelial dysfunction and immunothrombosis as key pathogenic mechanisms in COVID-19. Nat Rev Immunol. (2021) 21:319–29. doi: 10.1038/s41577-021-00536-9 PMC8023349 33824483

[45] Katsoulis O Toussaint M Jackson MM Mallia P Footitt J Mincham KT. Neutrophil extracellular traps promote immunopathogenesis of virus-induced COPD exacerbations. Nat Commun. (2024) 15(1):5766. doi: 10.1038/s41467-024-50197-0 38982052 PMC11233599

[46] Sandoval-Pérez A Berger RML Garaizar A Farr SE Brehm MA König G. DNA binds to a specific site of the adhesive blood-protein von Willebrand factor guided by electrostatic interactions. Nucleic Acids Res. (2020) 48:7333–44. doi: 10.1093/nar/gkaa466 PMC7367192 32496552

[47] Grässle S Huck V Pappelbaum KI Gorzelanny C Aponte-Santamaría C Baldauf C. von Willebrand factor directly interacts with DNA from neutrophil extracellular traps. Arterioscler Thromb Vasc Biol. (2014) 34:1382–9. doi: 10.1161/atvbaha.113.303016 24790143

[48] Kim SJ Carestia A McDonald B Zucoloto AZ Grosjean H Davis RP. Platelet-mediated NET release amplifies coagulopathy and drives lung pathology during severe influenza infection. Front Immunol. (2021) 12:772859. doi: 10.3389/fimmu.2021.772859 34858432 PMC8632260

[49] Merrill JT Erkan D Winakur J James JA. Emerging evidence of a COVID-19 thrombotic syndrome has treatment implications. (2020) 16(10):581–9. doi: 10.1038/s41584-020-0474-5 PMC7391481 32733003

[50] Chen F Chu C Wang X Yang C Deng Y Duan Z. Hesperetin attenuates sepsis-induced intestinal barrier injury by regulating neutrophil extracellular trap formation via the ROS/autophagy signaling pathway. Food Funct. (2023) 14:4213–27. doi: 10.1039/d2fo02707k 37067254

[51] Yang SC Chen PJ Chang SH Weng YT Chang FR Chang KY. Luteolin attenuates neutrophilic oxidative stress and inflammatory arthritis by inhibiting Raf1 activity. Biochem Pharmacol. (2018) 154:384–96. doi: 10.1016/j.bcp.2018.06.003 29883707

[52] Ma Q Chen R Zeng J Lei B Ye F Wu Q. Investigating the effects of Liushen Capsules (LS) on the metabolome of seasonal influenza: A randomized clinical trial. Front Pharmacol. (2022) 13:968182. doi: 10.3389/fphar.2022.968182 36034844 PMC9402892

[53] Wang C Cao B Liu QQ Zou ZQ Liang ZA Gu L. Oseltamivir compared with the Chinese traditional therapy maxingshigan-yinqiaosan in the treatment of H1N1 influenza: a randomized trial. Ann Intern Med. (2011) 155:217–25. doi: 10.7326/0003-4819-155-4-201108160-00005 21844547

[54] Fang J Li H Du W Yu P Guan YY Ma SY. Efficacy of early combination therapy with lianhuaqingwen and arbidol in moderate and severe COVID-19 patients: A retrospective cohort study. Front Pharmacol. (2020) 11:560209. doi: 10.3389/fphar.2020.560209 33071781 PMC7530276

