# Supplementary material for: Jing-Yin-Gu-Biao formula protects mice from postinfluenza Staphylococcus aureus infection by ameliorating acute lung injury and improving hypercoagulable state via inhibiting NETosis
Source: Front Immunol. 2025 Mar 11;16:1567522. doi: 10.3389/fimmu.2025.1567522 (PMC11933027; doi:10.3389/fimmu.2025.1567522)
Supplement: Supplementary file 1 [file DataSheet1.docx]

Supplementary Material

1. **Supplementary methods**

**1.1 Detection of the absorbed chemical constituents in the sera of mice**

Mice were administered with JYGBF (8 g/kg) or deionized water for 7 days, and the blood samples were collected from mice at 1 h after the last oral administration. Blood samples were placed at room temperature for 1 h and were centrifuged at 3,000 rpm for 25 min. Serum samples (400 μL) were added to 40 μL hydrochloric acid (2 mol/L). Subsequently, they were vortexed for 1 min and incubated for 15 min at 4 ℃, which was repeated 4 times. Then 1.6 mL acetonitrile was added to the mixture and vortexed for 5 min. The mixture was centrifuged at 12,000 rpm for 5 min at 4 ℃ and 1,800 μL supernatants were nitrogen dried. Then 150 μL of 80% methyl alcohol containing 10 μg/mL of internal standard were added to the dried samples and vortexed for 5 min. The samples were centrifuged at 12,000 rpm for 5 min at 4 ℃ and supernatants (120 μL) were used for LC/MS analysis as previously described [1].

JYGBF granules were mixed with beads and 500 μL of extraction solution that contained internal standards. The mixture was vortexed for 30 s, homogenized, and sonicated for 1 h at 4 ℃. Then the mixture was incubated for 1 h at -20 ℃ to precipitate proteins and was centrifuged at 12,000 rpm for 15 min at 4 ℃. The supernatants were used for LC/MS analysis as previously described [1].

# Supplementary Figures and Tables

## Supplementary Figure1


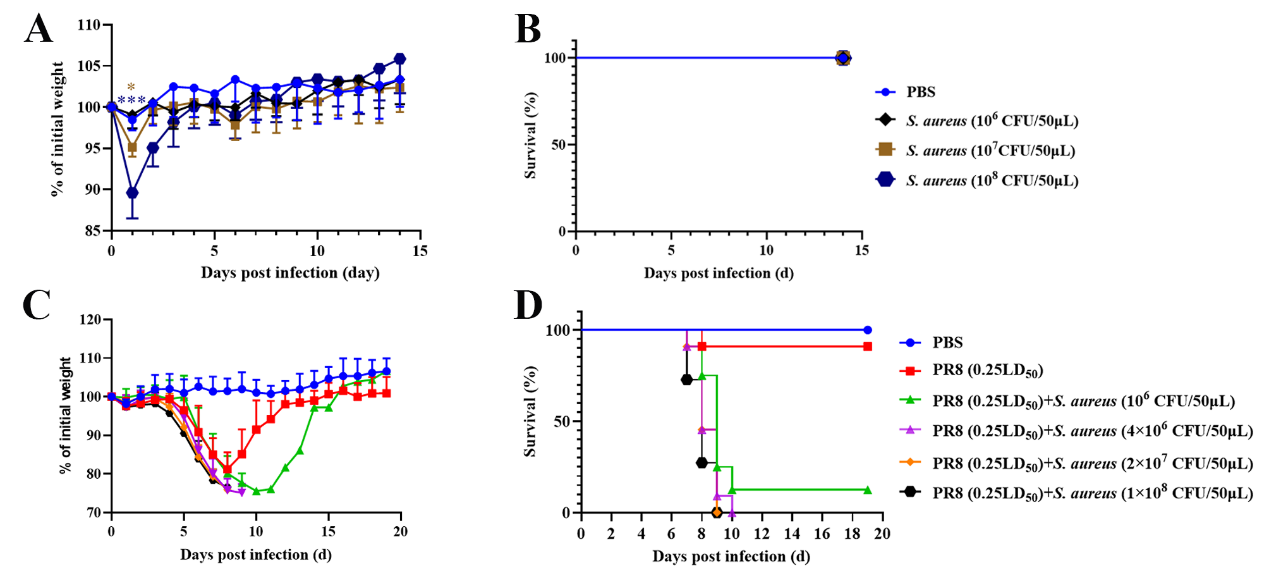


Fig. S1. The establishment of a mouse model of secondary *Staphylococcus aureus* (*S. aureus*) infection following influenza virus (PR8) infection. (A) The body weight change of mice infected by different CFU of *S. aureus*. (B) The survival rate of mice infected by different CFU of *S. aureus*. (C) The body weight change of mice infected by PR8 and different CFU of *S. aureus*. (D) The survival rate of mice infected by PR8 and different CFU of *S. aureus*.

## Supplementary Figure2


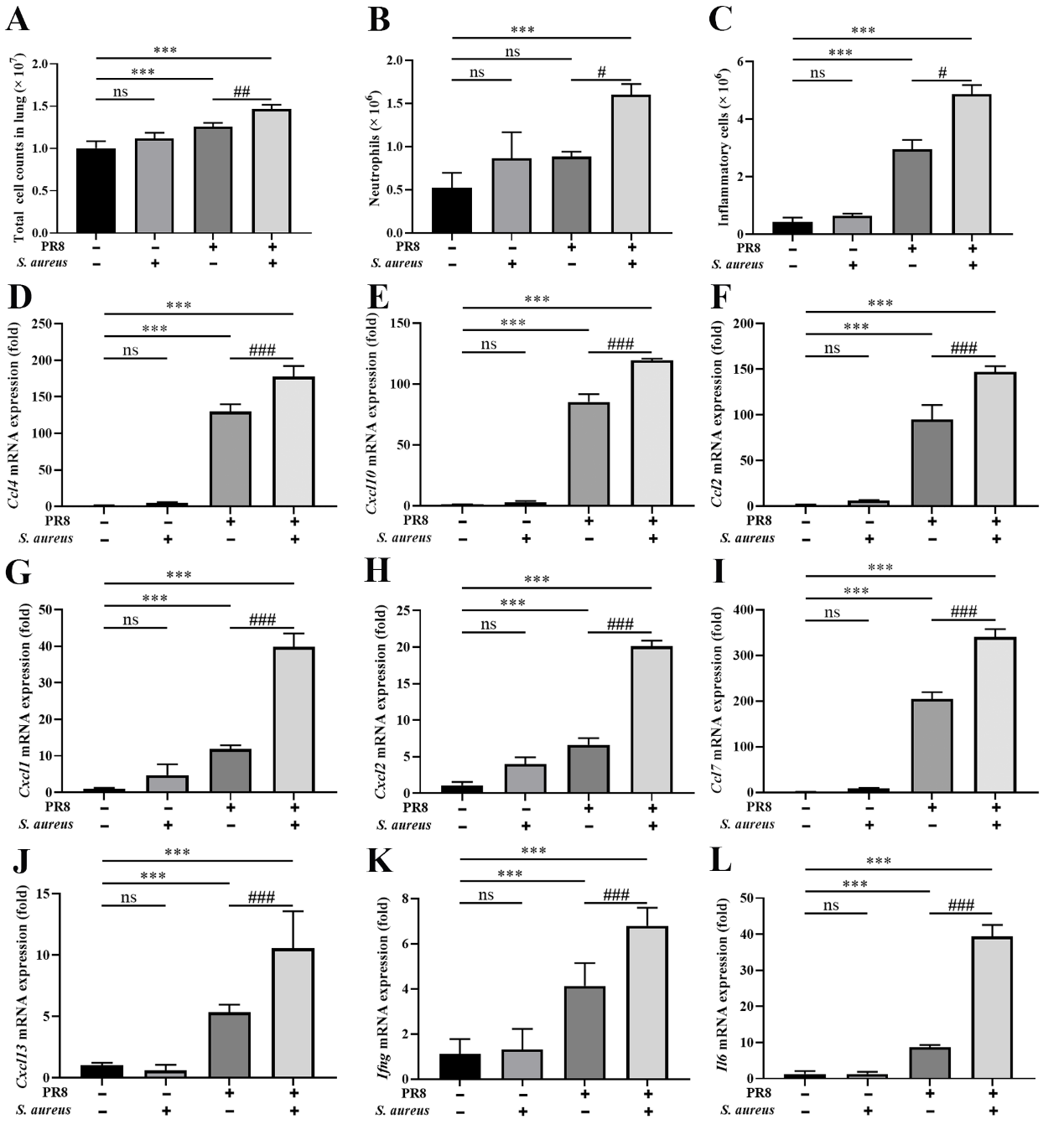


Fig. S2. Postinfluenza *S. aureus* infection leads to a more aggressive immune response. (A-C) The total cell counts, neutrophils and inflammatory monocytes in lungs of PBS group, PR8 group (0.25 LD_50_), *S. aureus* group (1 × 10^6^ CFU/50 μL) and postinfluenza *S. aureus* infection group. (D-L) The mRNA expression of *Ccl4*, *Cxcl10*, *Ccl2*, *Cxcl1*, *Cxcl2*, *Ccl7*, *Cxcl13*, *Ifng* and *Il6* in lungs of PBS group, PR8 group (0.25 LD_50_), *S. aureus* group (1 × 10^6^ CFU/50 μL) and postinfluenza *S. aureus* infection group.

## Supplementary Figure3
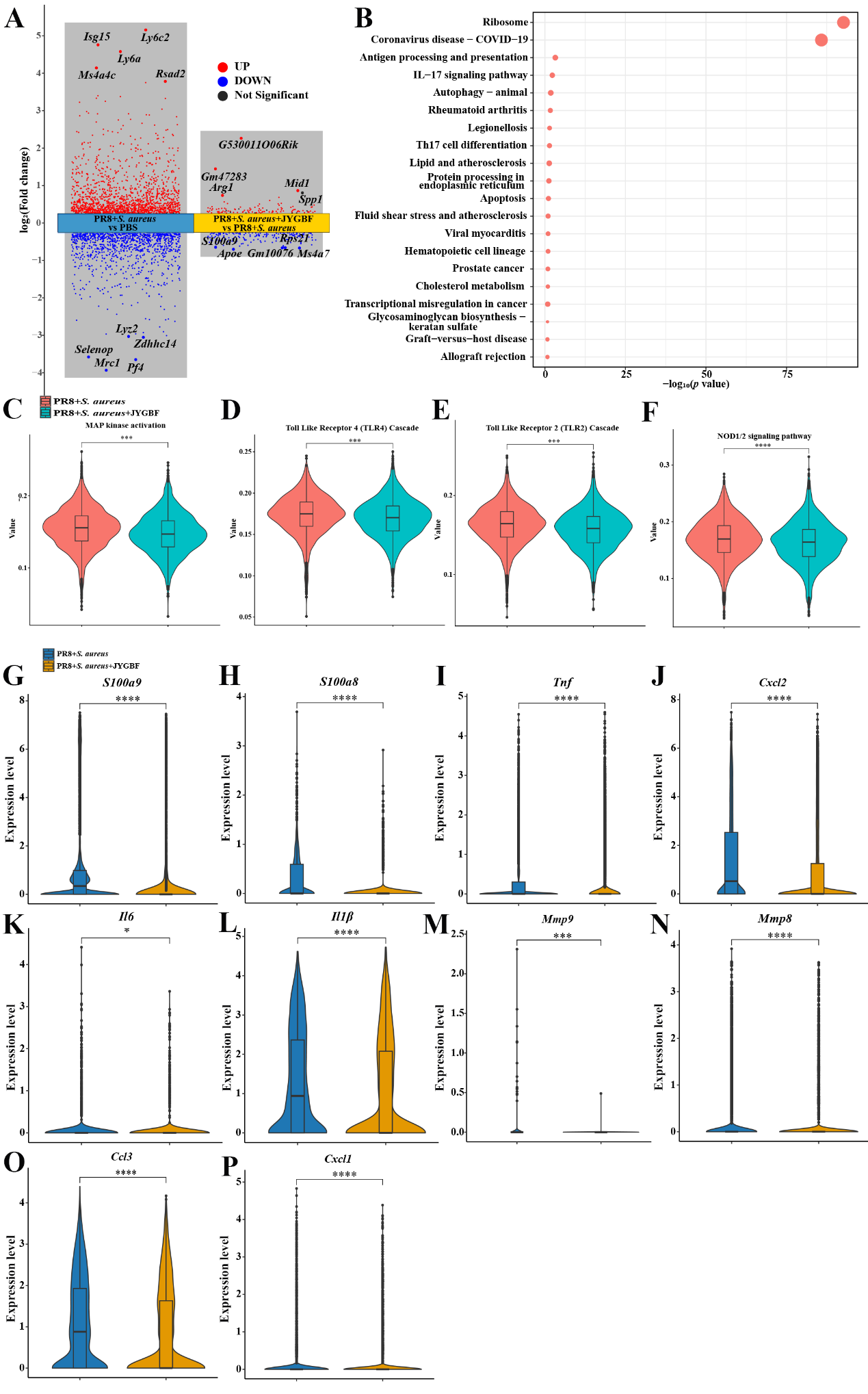


Fig. S3. ScRNA-seq data of IMs. (A) Upregulated genes and downregulated genes in postinfluenza *S. aureus* infection and JYGBF-treated group. (B) KEGG analysis of downregulated genes. (C-F) GSVA data of MAPK, TLR4, TLR2 and NOD1/2. (G-P) The relative mRNA expression of *S100a9*, *S100a8*, *Tnf*, *Cxcl2*, *Il6*, *Il1b*, *Mmp9*, *Mmp8*, *Ccl3* and *Cxcl1* in IMs.

## Supplementary Figure4


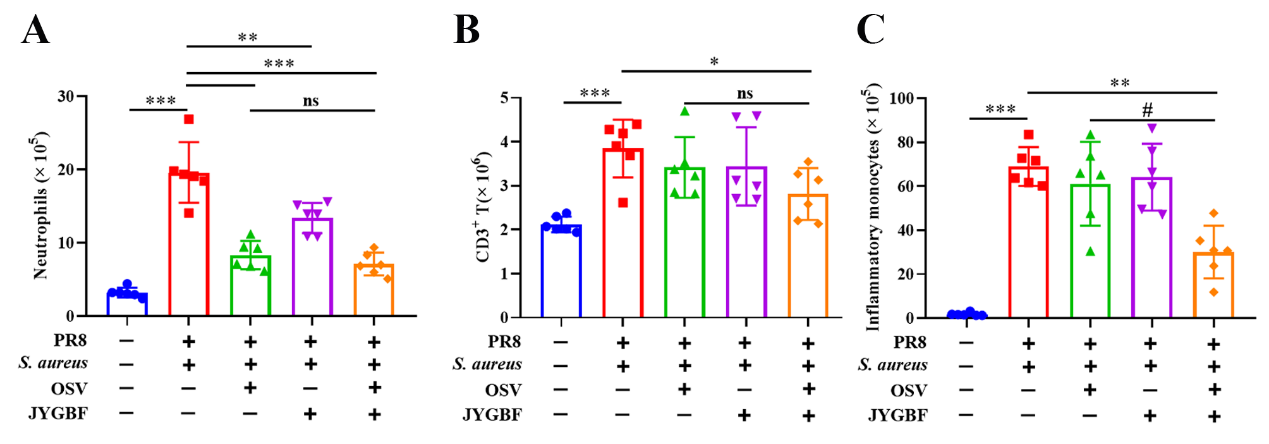


Fig. S4. Flow cytometric analysis of immune cells in lungs. (A-C) The cell number of neutrophils, CD3^+^ T cells and inflammatory monocytes in lungs of mice.

## Supplementary Figure5


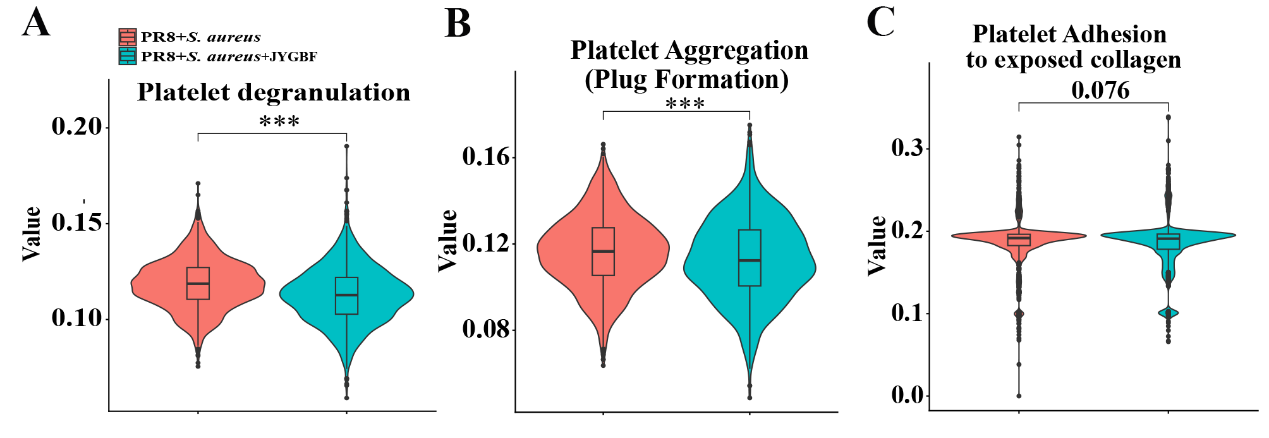


Fig.S5. JYGBF inhibited the activation of platelets. (A-C) GSVA data of platelet degranulation, platelet aggregation, and platelet adhesion to exposed collagen.

## Supplementary Figure6


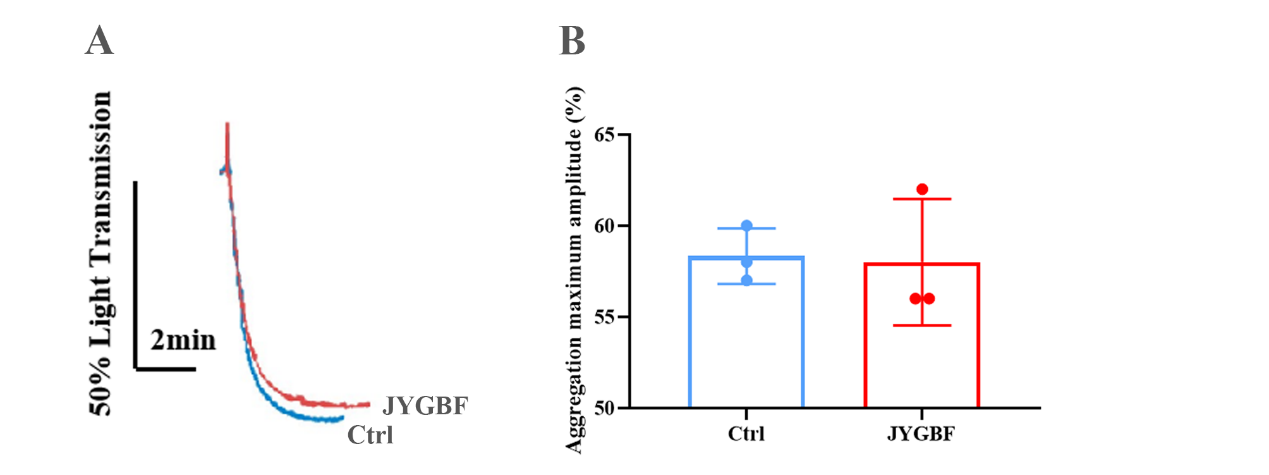


Fig. S6. JYGBF could not inhibit platelet aggregation *in vitro*. (A) Platelet aggregation induced by ADP (5 μM) with the treatment of control sera or JYGBF-containing sera. Platelets were obtained from SD rats. (B) The percentage of aggregation maximum amplitude, data were shown as mean ± SD.

## Supplementary Figure7


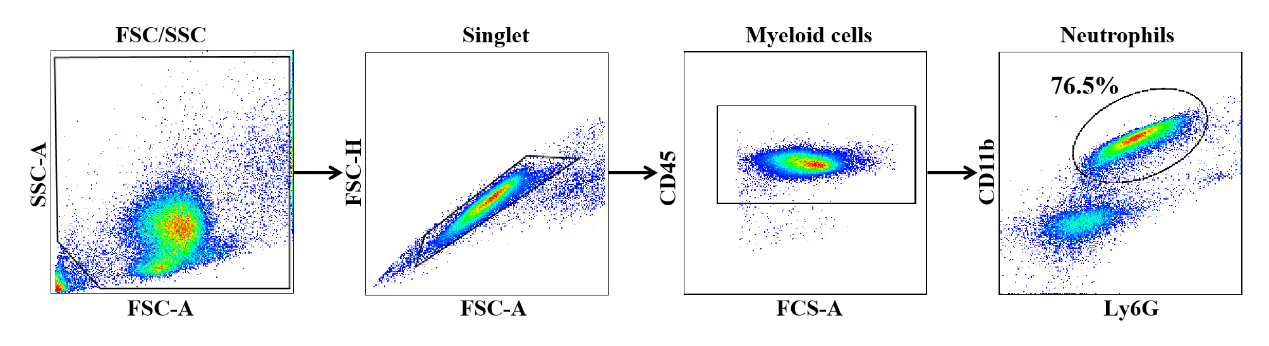


Fig. S7. The analytical strategies of neutrophils isolated from bone marrow. The purity of neutrophil was typically > 76%, which was determined as CD11b^+^ Ly6G^+^ cells by Flow cytometry.

## Supplementary Figure8


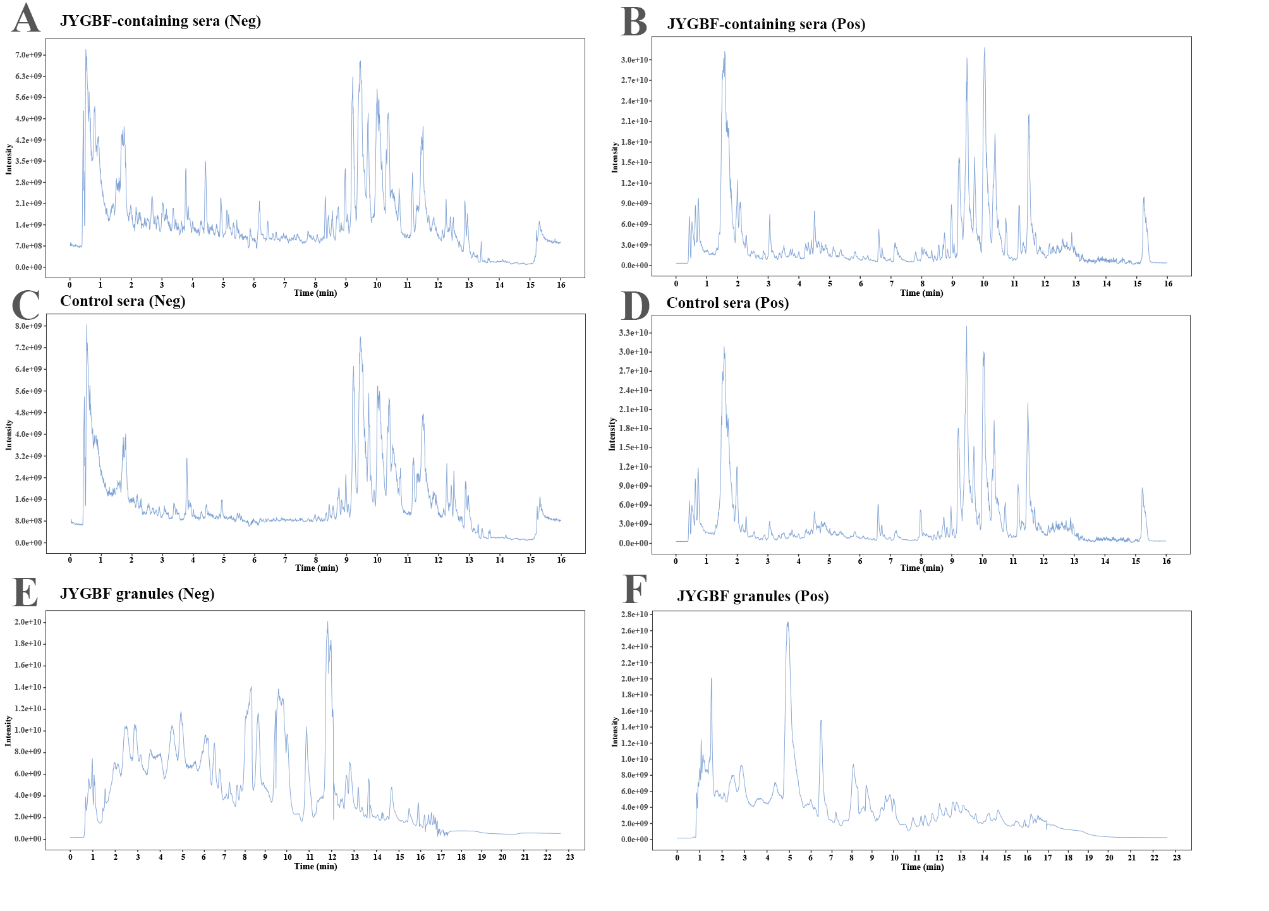


Fig. S8. Total ion chromatogram (TIC) of JYGBF-containing sera and control sera by UHPLC-QE-MS. (A-B) Negative and positive ion modes of JYGBF-containing sera. (C-D) Negative and positive ion modes of control sera. (E-F) Negative and positive ion modes of JYGBF granules.

**1.9 Supplementary Table2** Primer sequences of target genes

| **Target Gene** | **Primer Sequence** |
| --- | --- |
| mβ-Actin | 5’- AGTGTGACGTTGACATCCGT -3’ (F)  5’- GCAGCTCAGTAACAGTCCGC -3’ (R) |
| m*Il1b* | 5’ - GGTGTGTGACGTTCCCATTAGAC - 3’ (F)  5’ - CATGGAGAATATCACTTGTTGGTTGA - 3’ (R) |
| m*Ccl2* | 5’- GCATCCACGTGTTGGCTCA -3’ (F)  5’- CTCCAGCCTACTCATTGGGATCA -3’ (R) |
| m*Cxcl2* | 5’- GGTTGACTTCAAGAACATCCAG -3’ (F)  5’- TTGAGAGTGGCTATGACTTCTG -3’ (R) |
| m*Tnf* | 5’- AAGCCTGTAGCCCACGTCGTA -3’ (F)  5’- GGCACCACTAGTTGGTTGTCTTTG -3’ (R) |

**1.10 Supplementary Table3** A total of 30 compounds identified in the sera of mice after 7 consecutive days’ gavage of JYGBF

| NO. | Name | InChIKey | Formula | mzmed | rtmed (s) | type |
| --- | --- | --- | --- | --- | --- | --- |
| 1 | Gentisic acid | WXTMDXOMEHJXQO-UHFFFAOYSA-N | C7H6O4 | 153.0 | 57.8 | neg |
| 2 | Glabrolide | SSHDNSCEQSPWIM-FVTWEACWSA-N | C30H44O4 | 469.3 | 328.0 | pos |
| 3 | Glycyrrhizic acid | LPLVUJXQOOQHMX-UHFFFAOYSA-N | C42H62O16 | 821.4 | 353.2 | neg |
| 4 | Rosmarinic acid | DOUMFZQKYFQNTF-WUTVXBCWSA-N | C18H16O8 | 359.1 | 149.7 | neg |
| 5 | Secoxyloganin | MQLSOVRLZHTATK-PEYNGXJCSA-N | C17H24O11 | 427.1 | 72.6 | pos |
| 6 | Methylnissolin | UOVGCLXUTLXAEC-WFASDCNBSA-N | C17H16O5 | 301.1 | 217.8 | pos |
| 7 | Formononetin | HKQYGTCOTHHOMP-UHFFFAOYSA-N | C16H12O4 | 269.1 | 307.2 | pos |
| 8 | Isovitexin | MYXNWGACZJSMBT-VJXVFPJBSA-N | C21H20O10 | 431.1 | 99.2 | neg |
| 9 | Acacetin | DANYIYRPLHHOCZ-UHFFFAOYSA-N | C16H12O5 | 283.1 | 201.8 | neg |
| 10 | Cimifugin | ATDBDSBKYKMRGZ-ZDUSSCGKSA-N | C16H18O6 | 307.1 | 125.8 | pos |
| 11 | Wogonin | XLTFNNCXVBYBSX-UHFFFAOYSA-N | C16H12O5 | 285.1 | 3.2 | pos |
| 12 | Licochalcone B | DRDRYGIIYOPBBZ-XBXARRHUSA-N | C16H14O5 | 287.1 | 321.6 | pos |
| 13 | Licochalcone E | SWPKMTGYQGHLJS-RNVIBTMRSA-N | C21H22O4 | 337.1 | 268.9 | neg |
| 14 | Isoliquiritigenin | DXDRHHKMWQZJHT-FPYGCLRLSA-N | C15H12O4 | 257.1 | 188.4 | pos |
| 15 | Liquiritigenin | FURUXTVZLHCCNA-AWEZNQCLSA-N | C15H12O4 | 257.1 | 99.8 | pos |
| 16 | Isorhamnetin | IZQSVPBOUDKVDZ-UHFFFAOYSA-N | C16H12O7 | 315.1 | 267.0 | neg |
| 17 | Ononin | MGJLSBDCWOSMHL-MIUGBVLSSA-N | C22H22O9 | 431.1 | 189.5 | pos |
| 18 | Pogostone | AJFJTORMMHWKFW-UHFFFAOYSA-N | C12H16O4 | 225.1 | 492.1 | pos |
| 19 | Luteolin | IQPNAANSBPBGFQ-UHFFFAOYSA-N | C15H10O6 | 285.0 | 202.2 | neg |
| 20 | Chrysoeriol | SCZVLDHREVKTSH-UHFFFAOYSA-N | C16H12O6 | 301.1 | 203.9 | pos |
| 21 | Hesperetin | AIONOLUJZLIMTK-UHFFFAOYSA-N | C16H14O6 | 301.1 | 260.5 | neg |
| 22 | Eupatilin | DRRWBCNQOKKKOL-UHFFFAOYSA-N | C18H16O7 | 345.1 | 414.5 | pos |
| 23 | Pelargonic acid | FBUKVWPVBMHYJY-UHFFFAOYSA-N | C9H18O2 | 157.1 | 341.7 | neg |
| 24 | Caprylic acid | WWZKQHOCKIZLMA-UHFFFAOYSA-N | C8H16O2 | 143.1 | 277.0 | neg |
| 25 | Erucamide | UAUDZVJPLUQNMU-KTKRTIGZSA-N | C22H43NO | 338.3 | 772.3 | pos |
| 26 | 5-Hydroxymethylfurfural | NOEGNKMFWQHSLB-UHFFFAOYSA-N | C6H6O3 | 109.0 | 22.1 | pos |
| 27 | Isoliquiritin | YNWXJFQOCHMPCK-LXGDFETPSA-N | C21H22O9 | 417.1 | 99.5 | neg |
| 28 | Indigo | QQILFGKZUJYXGS-UHFFFAOYSA-N | C16H10O2N2 | 263.1 | 374.5 | pos |
| 29 | Isatin | JXDYKVIHCLTXOP-UHFFFAOYSA-N | C8H5NO2 | 148.0 | 81.3 | pos |
| 30 | 3-Hydroxy-2-Methylpyridine | AQSRRZGQRFFFGS-UHFFFAOYSA-N | C6H7NO | 110.1 | 3.7 | pos |

Reference:

[1] X. Wu, L. Xu, G. Xu, Y. Xu, H. Liu, Y. Hu, X. Ye, Q. Huang, C. Tang, N. Duan, X. Chen, X.D. Yang, W. Zhang, Y. Zheng, Fei-yan-qing-hua decoction exerts an anti-inflammatory role during influenza by inhibiting the infiltration of macrophages and neutrophils through NF-κB and p38 MAPK pathways, J Ethnopharmacol 337(Pt 2) (2025) 118846.
